# microRNA-1271 impedes the development of prostate cancer by downregulating PES1 and upregulating ERβ

**DOI:** 10.1186/s12967-020-02349-1

**Published:** 2020-05-24

**Authors:** Zhenming Jiang, Yuxi Zhang, Xi Chen, Yan Wang, Pingeng Wu, Chengzhang Wu, Dong Chen

**Affiliations:** 1 Department of Urology, The First Hospital of China Medical University, No. 155 Nanjing North Street, Heping District, Shenyang, 110001 Liaoning Province People’s Republic of China; 2 Department of Urology, People’s Hospital of Datong Hui and Tu Autonomous County, Xining, 810100 People’s Republic of China; 3 Department of Pharmacy, The First Hospital of China Medical University, Shenyang, 110001 People’s Republic of China; 4 Department of Pathology, The First Hospital and College of Basic Medical Sciences, China Medical University, Shenyang, 110001 People’s Republic of China; 5 Central Lab, The First Hospital of China Medical University, Shenyang, 110001 People’s Republic of China

**Keywords:** Prostate cancer, microRNA-1271, Pescadillo homolog 1, Estrogen receptor β

## Abstract

**Background:**

As a nucleolar protein associated with ribosome biogenesis, pescadillo homolog 1 (PES1) has been reported to participate in the development of many cancers. However, its role in prostate cancer is not clearly defined. Therefore, the aim of this study is to explore the effects and the specific mechanism of PES1 in prostate cancer.

**Methods:**

A microarray-based analysis was performed to analyze differentially expressed genes (DEGs) between prostate cancer and normal samples. Next, the interaction between PES1 and microRNA-1271 (miR-1271) was investigated using bioinformatics analysis in combination with dual-luciferase reporter gene assay. The expression of miR-1271 in prostate cancer cells and tissues was determined using RT-qPCR. Its effects on downstream estrogen receptor β (ERβ) signaling pathway were further examined. Moreover, we analyzed whether miR-1271 affects proliferation, apoptosis, migration and invasion of prostate cancer cells by EdU assay, flow cytometry, and Transwell assay. Lastly, a prostate cancer mouse model was conducted to measure their roles in the tumor growth.

**Results:**

PES1 was identified as a prostate cancer-related DEG and found to be upregulated in prostate cancer. miR-1271, which was poorly expressed in both cells and tissues of prostate cancer, can specifically bind to PES1. Additionally, overexpression of miR-1271 activated the ERβ signaling pathway. Overexpression of miR-1271 or depletion of PES1 inhibited prostate cancer cell proliferation, migration and invasion, promoted apoptosis in vitro and suppressed tumor growth in vivo.

**Conclusions:**

Taken together, overexpression of miR-1271 downregulates PES1 to activate the ERβ signaling pathway, leading to the delayed prostate cancer development. Our data highlights the potential of miR-1271 as a novel biomarker for the treatment of prostate cancer.

## Background

Prostate cancer ranks second among the prevalent cancers in males and approximately 1.1 million people are diagnosed with prostate cancer annually in the world [1], with an increasing incidence rate in most Asian countries due to various factors such as economic development and food consumption [2]. Prostate cancer can be induced by numerous risk factors, including smoking, drinking, obesity, as well as occupational exposure to pesticides and cadmium [3]. Further, prostate cancer is defined as a heterogeneous disease characterized by variable perspectives and there are several approaches to treat this disease, such as active surveillance, radiotherapy. However, defining the best strategy for prostate cancer patients is still a challenge [4, 5]. Hence, it is important to provide more future directions for prostate cancer treatment.

Our microarray-based analysis revealed that pescadillo homolog 1 (PES1) gene was differentially expressed in prostate cancer and was involved in the prostate cancer development. As a nucleolar protein, PES plays an essential role in the viability of yeast as well as higher eukaryotes and participates in multiple cellular development, including cell proliferation, ribosome biogenesis, and DNA replication [6]. PES1 has been reported to be highly expressed in gastric cancer and downregulation of PES1 suppresses the progression of gastric cancer, thus serving as a critical biomarker for the diagnosis of gastric cancer [7]. PES1 gene is upregulated in the microvesicles of prostate cancer cells and involved in the pathogenesis of prostate cancer [8].

As a kind of short non-coding RNA downregulating the target mRNA expression, microRNAs (miRNAs) are widely known to be involved in diverse cancer processes and accepted as potential prognostic and diagnostic biomarkers for patients suffering from cancer [9]. miR-1271 is found decreased in pancreatic cancer tissues [10]. miR-1271 is also poorly expressed in prostate cancer tissues and can function as a biomarker for management of prostate cancer patients [11]. Based on the bioinformatic prediction and dual-luciferase reporter gene assay in the current study, PES1 is a potent target gene of miR-1271 in prostate cancer. PES1 mediates the balance between estrogen receptor (ER)β and ERα in tumor growth of estrogen-provoked breast cancer [12]. ERβ, encoded by chromosome locus 14q22-24, belongs to the nuclear receptor supergroup and expression of this gene can be found in luminal epithelial cells and the stromal cells of the prostate [13]. A number of studies have revealed the suppressive effect of ERβ on the development of prostate cancer and that ERβ acts as a marker for the management of prostate cancer [14, 15]. Based on these findings and analyses, we hypothesized that the interaction among miR-1271, PES1 and ERβ may participate in the progression of prostate cancer. Therefore, the present study was designed to uncover the specific mechanisms of these genes in prostate cancer via both in vitro and in vivo assays.

## Methods

### Ethics statement

This study was approved by the Ethics Committee of The First Hospital of China Medical University performed in strict accordance with the *Declaration of Helsinki*. All participants or their relatives signed informed consent documentation. Animal experiments strictly adhered to the principle to minimize the pain, suffering and discomfort to the experimental animals.

### Patient enrollment

Prostate cancer tissues were collected through fine-needle biopsy from 54 patients (aged from 43 to 82 years old with a mean age of 63.24 ± 6.52 years) who were pathologically diagnosed as prostate cancer at the Department of Urology in The First Hospital of China Medical University from June, 2017 to December, 2017. The enrolled patients did not receive any treatment before blood test. The patients suffered from hypertension, acute infection, tuberculosis, and diabetes mellitus were excluded. According to the Gleason score of histopathological classification formulated by World Health Organization in 2003 [16], different patients had various pathological scores (19 patients with 6 scores, 18 patients with 7 scores, 9 patients with 8 scores, and 8 patients with 9 scores). As for tumor-nodes-metastasis (TNM) stages, 3 patients were at T_1b_N_0_M_0_ stage, 17 patients were at T_1c_N_0_M_0_, 15 patients were at T_2a_N_0_M_0_, 11 patients were at T_2b_N_0_M_0_, and 8 patients were at T_2c_N_0_M_0_. A total of 15 patients pathologically diagnosed as benign prostatic hyperplasia through transurethral resection of prostate or prostate biopsy were selected as controls. These patients did not experience other malignancies, coronary heart disease, and diabetes mellitus.

### Dual-luciferase reporter gene assay

The plasmid vectors pGL3-basic (Shanghai Realgene Co., Ltd., Shanghai, China) harboring PES1-3′-untranslated region (UTR)-wild type (Wt) and PES-3′UTR-Mutant (Mut) were constructed and transfected with miR-1271 mimic or NC plasmids into cells. After 48 h of transfection, the luciferase activity of samples was detected using the dual-luciferase reporter gene detection kits (E1960, Promega Corp., Madison, Wisconsin, USA).

### Cell harvest and treatment

Four prostate cancer cell lines (LNCaP, 22Rv1, PC-3, and DU145) and one normal prostatic epithelial cell line (RWPE-1) purchased from American Type Culture Collection (Manassas, VA, USA) were cultured in Dulbecco’s modified Eagle’s medium (DMEM; Gibco, Carlsbad, California, USA) containing 10% fetal bovine serum (FBS) (Hangzhou Sijiqing Company, Zhejiang, China) and 1% penicillin (100 U/L)-streptomycin (100 mg/L) (Gibco, Carlsbad, California, USA) at 37 °C with 5% CO_2_. In the following experiments, the cells were treated with miR-1271 mimic, miR-1271 inhibitor, short hairpin RNA (sh)-PES1, pyrazolo [1,5-a] pyrimidine (PHTPP) (ERβ antagonist), or their respective negative controls (NCs) (NC mimic, NC inhibitor, sh-NC, dimethyl sulfoxide [DMSO]) based on the manufacturer’s guide. Transient transfection was performed using Lipofectamine 2000 reagents (Invitrogen, USA). Each transfection sequence is listed in Additional file 1: Table S1.

### Reverse transcription quantitative polymerase chain reaction (RT-qPCR)

Total RNA was extracted from the prostate cancer cell lines using the RNA extraction kits (Invitrogen, Inc., Carlsbad, CA, USA). The designed primers (Table 1) were synthesized by Takara (Tokyo, Japan). The extracted RNA was reversely transcribed into complementary DNA based on the protocol of PrimeScript RT kits. RT-qPCR was conducted using the ABI PRISM^®^ 7300 system. U6 was used as the internal control for miR-1271 and glyceraldehyde-3-phosphate dehydrogenase (GAPDH) for PES1. The transcription level of the target genes was finally calculated using the 2^−ΔΔCt^ method.

**Table 1:** Primer sequences for RT-qPCR

Gene	Primer sequence (5′-3′)
miR-1271	F: 5′-CTAGACGTCCAGATTGAATAGAC-3′
	R: 5′-GTCCGAGCTTGGTCAGAATG-3′
PES1	F: 5′-GAGGCTCACAGTCAATGAATCGTC-3′
	R: 5′-AAACGTTCGGGCTGCTGTAGA-3′
U6	F: 5′-AGCCCGCACTCAGAACATC-3′
	R: 5′-GCCACCAAGACAATCATCC-3′
GAPDH	F: 5′-GCACCGTCAAGGCTGAGAAC-3′
	R: 5′-TGGTGAAGACGCCAGTGGA-3′

RT-qPCR, reverse transcription quantitative polymerase chain reaction; PES1, pescadillo homolog 1; GAPDH, glyceraldehyde-3-phosphate dehydrogenase; F, forward; R, reverse

### Western blot analysis

Total protein was extracted from cell lysates. After separation using 10% sodium dodecyl sulfate polyacrylamide gel electrophoresis, the protein was transferred onto the polyvinylidene fluoride membrane using a wet transfer method. Subsequently, the membrane was blocked with 5% skim milk and probed with the primary antibodies, followed by further incubation with secondary antibody labeled by horseradish peroxidase. The membrane was developed and then visualized using the enhanced chemiluminescence detection kits (BB-3501, Amersham, Little Chalfont, UK). Images were captured using the Bio-Rad image analysis system (Bio-Rad, Inc., Hercules, CA, USA) and the results were analyzed using the Quantity One v4.6.2 software. The relative protein level was expressed as the ratio of gray value of target bands to that of β-actin band. All primary antibodies were supplied by Abcam Inc., Cambridge, UK: GAPDH (ab8245; 1: 1000), total caspase-3 (ab13847, 1: 1000), c-caspase-3 (ab32150, 1 : 1000), c-caspase-9 (ab2324, 1 : 1000), ERα (ab241587, 1 : 5000), ERβ (ab268053, 1 : 5000), proliferating cell nuclear antigen (PCNA; ab18197, 1 : 1000), PES1 (ab72539, 1 : 5000), Ki67 (209897, 1 : 5000), matrix metallopeptidase (MMP)-2 (ab37150, 1 : 1000), and MMP-9 (ab38898, 1 : 1000).

### 5-ethynyl-2′-deoxyuridine (EdU) assay

Cells were incubated with the addition of EdU solution (a mixture of culture medium and EdU solution at 1000 : 1) at room temperature for 2 h, fixed with 40 g/L polyformaldehyde for 30 min, incubated with glycine solution for 8 min, and rinsed with phosphate-buffered saline (PBS) containing 0.5% Triton X-100. After stained with Apollo^®^ reaction liquid and Hoechst 3334 for 30 min and 20 min respectively at room temperature in the dark, the cells were observed under a fluorescence microscope. Images were then captured under red light at an excitation wavelength of 550 nm and under purple light at an excitation wavelength of 350 nm. The cells with red fluorescence were proliferative cells and cells with blue fluorescence were total cells. Next, the number of EdU-stained cells (proliferative cells) and Hoechst 3334-stained cells (total cells) was counted in 3 randomly selected fields under the magnitude of 400. The cell proliferation rate = the number of proliferative cells/the number of total cells × 100%.

### Annexin V-FITC/PI staining and flow cytometry

After 48 h of transfection, cells were washed with PBS and detached by trypsinization. Cells were harvested at a density of 1 × 10^6^ cells/mL by centrifugation at 1000 r/min at 4 °C for 5 min. Next, the cells were resuspended in 400 μL 1 × binding buffer with the cell concentration adjusted to 0.5–1 × 10^6^ cells/mL. With the addition of 5 μL Annexin V-fluorescein isothiocyanate in cell suspension, cells were incubated at 4 °C for 15 min avoiding exposure to light. Moreover, the cells were incubated at 4 °C for 15 min under dark conditions with 5 μL propidium iodide. Cell apoptosis rate was measured using a flow cytometer. Log FL1-Log FL2 (X axis-Y axis) dual-parameter dot plots were drawn and the data were analyzed.

### Transwell migration and invasion assays

Transwell chamber with 0.8 μm aperture diameter (Corning, New York, USA) was employed for cell migration assays and matrigel-coated Transwell chamber with 0.8 μm aperture diameter (BD, Franklin Lakes, NJ, USA) was applied for cell invasion detection. In short, cells were starved with serum-free DMEM for 24 h. The basolateral Transwell chamber (Corning, New York, USA) was cultured with serum-free DMEM at 37 °C for 1 h. After the detachment, cells were resuspended in serum-free DMEM and diluted to 3 × 10^5^ cells/mL. Afterwards, 100 μL cells were added to the upper Transwell chamber and 600 μL DMEM supplemented with 10% FBS (chemokine) was added to the basolateral Transwell chamber, followed by incubation for 24 h. Subsequently, the cells that transferred into the basolateral chamber were subjected to 0.1% crystal violet staining for 10 min. The cells were finally observed in six randomly selected fields under an inverted microscope (Olympus Optical Co., Ltd, Tokyo, Japan) and images were obtained.

### Xenograft tumor in nude mice

A total of 84 specific pathogen-free male nude mice (aged 6 weeks and weighing 23–27 g) were purchased from Shanghai SLAC Laboratory Animal Co., Ltd. (Shanghai Laboratory Animal Center, Chinese Academy of Science, Shanghai, China). The nude mice were raised at constant temperature (25–27 °C) and constant humidity (45–50%). The cells transfected with sh-PES1, miR-1271 agomir, miR-1271 antagomir, PHTPP (10 μL of 1 × 10^−2^ M PHTPP per mouse [17], or their respective NCs (sh-NC, NC agomir, NC antagomir, DMSO) were resuspended in 50% Matrigel (BD Biosciences, Bedford, MA, USA) at a density of 2 × 10^6^ cells/mL. The transfected cells were used to construct stable cell line to prepare cell suspension, 0.2 mL of which (containing 4 × 10^5^ cells) was then subcutaneously injected into each nude mouse at left axilla. miR-1271 antagomir, miR-1271 agomir and their separate controls were Shanghai GenePharma Co. Ltd. (Shanghai, China). The tumor volume was measured every 7 days and the tumor growth curve was plotted. The nude mice were euthanized and the tumor tissues were extracted and weighed.

### Statistical analysis

The SPSS 21.0 statistical software (IBM Corp., Armonk, NY, USA) was used to analyze data in our study. Measurement data were presented as mean ± standard deviation. Data between two groups were analyzed using independent sample *t* test. The normal distribution was evaluated using the Kolmogorov–Smirnov test, with homogeneity of variance tested. Comparisons of data obeying normal distribution and homogeneity of variance among multiple groups were conducted using one-way analysis of variance (ANOVA), followed by Tukey’s post hoc tests with corrections for multiple comparisons. Variables at different time points were analyzed by repeated measures ANOVA with Bonferroni’s post hoc tests. Mann–Whitney U (non-parametric) test was used for data with skewed distribution or defect variances. Pearson’s correlation coefficient was used for analyzing the correlation between miR-1271 expression and Gleason scoring. The level of significance (*p* value) was set to 0.05.

## Results

### Analysis of microarray data from on-line databases

**Fig. 1:**
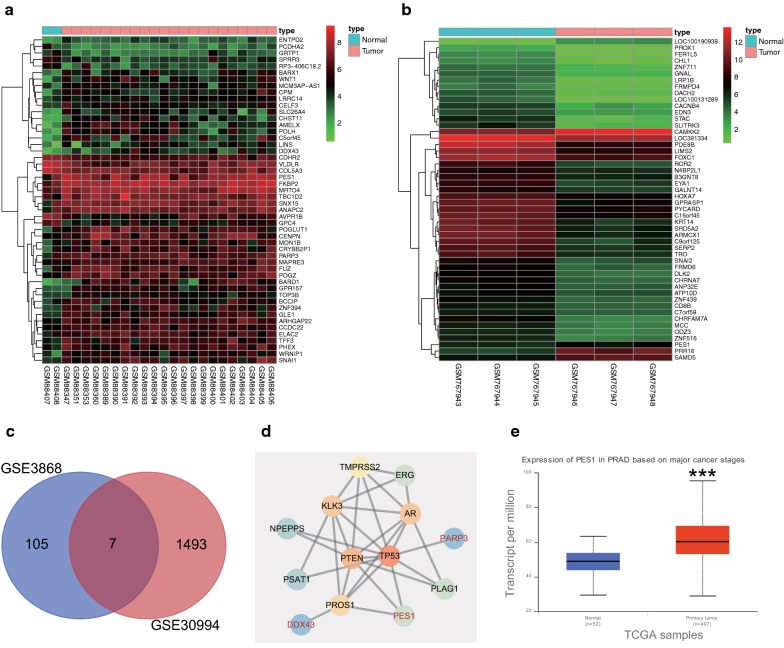
Analysis of microarray data from on-line databases. A-B, Expression heatmaps of the DEGs from the datasets GSE3868 (**a**) and GSE30994 (**b**), in which the X axis refers to sample number, and the Y axis refers to gene names; the left dendrogram represents gene expression cluster; each square represents the expression of a gene in each sample; the upper right squares refer to color scale. **c** Venn diagram showing the DEGs from the datasets GSE3868 (the left circle) and GSE30994 (the right circle); the intersection showing the overlapped DEGs in the two datasets. **d** Gene interaction network of 7 DEGs retrieved from the expression datasets and 10 known prostate cancer-related genes obtained from the DisGeNET database; each circle represents a gene and the lines between the circles denote the interaction between genes. **e** Expression of PES1 in the TCGA database, in which the X axis refers to sample type, and the Y axis represents gene expression; the left box plot denotes the expression of PES1 in normal samples and the right box plot shows the expression of PES1 in prostate cancer samples. ***p < 0.001, vs. normal samples

Microarray-based analysis was performed to screen the differentially expressed genes (DEGs) associated with prostate cancer. Two datasets related to prostate cancer (GSE3868 and GSE30994) were retrieved from the Gene Expression Omnibus (GEO) database. Through differential analysis of the gene expression in prostate cancer samples and normal samples, 224 and 3000 DEGs were obtained from GSE3868 and GSE30994 databases respectively. The heatmap generated from 50 DEGs from these two expression datasets were constructed, respectively (Fig. 1a, b). In order to further screen prostate cancer-related DEGs, the top 50% DEGs from the above two datasets were subjected to Venn analysis, which revealed 7 DEGs in the intersection of the results (Fig. 1c). The DisGeNET database was used to retrieve the known prostate cancer-related genes, 10 of which with the highest score and 7 intersected DEGs were selected to construct the gene interaction network (Fig. 1d). The results revealed that among 7 DEGs, only PES1, PARP3, and DDX43 were in the gene interaction network and PES gene was correlated to such core genes as TP53 and PTEN. Among PES1, PARP3, and DDX43 genes, PES1 was at the hub position in the gene interaction network. Further analysis of the prostate cancer datasets in The Cancer Genome Atlas (TCGA) indicated that PES1 gene expression was greatly upregulated in prostate cancer samples (Fig. 1e), which was in consistent with the gene expression in the prostate cancer-related expression datasets. Collectively, PES1 might play an important role in the development of prostate cancer.

### Silencing of PES1 inhibits the development of prostate cancer both in vivo and in vitro

**Fig. 2:**
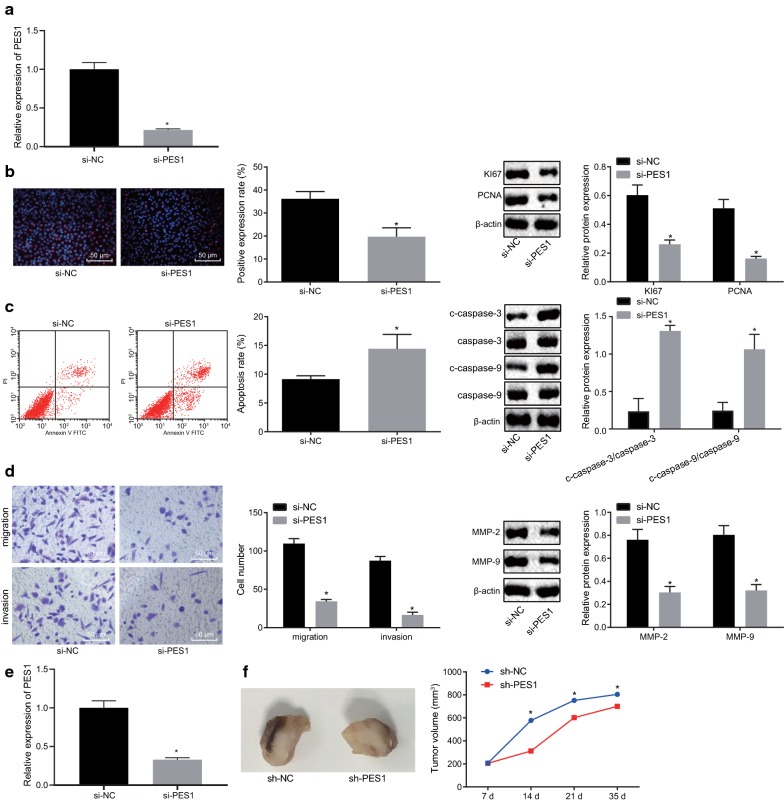
Silencing of PES1 inhibits the development of prostate cancer in vivo and in vitro. **a** PES1 mRNA expression in PC-3 cells detected by RT-qPCR. **b** Images of EdU assay (200×), positive rate of prostate cancer PC-3 cells and the expression of Ki67 and PCNA in response to si-NC or si-PES1 detected by EdU assay and Western blot analysis. **c** PC-3 cell apoptosis and protein expression of c-caspase-3/caspase-3 and c-caspase-9/caspase-9 in response to si-NC or si-PES1, as measured using flow cytometry and Western blot analysis. **d** PC-3 cell migration and invasion (200×) and protein expression of MMP-2 and MMP-9 in response to si-NC or si-PES1, as measured using Transwell assay and Western blot analysis. **e** PES1 mRNA expression in mouse tumors detected by RT-qPCR; **f** representative tumors and tumor growth rate of mice bearing human prostate cancer cell xenografts in response to sh-NC or sh-PES1 treatment (n = 6 for each group). *p < 0.05, vs. treatment with si-NC or sh-NC. Data obtained from three independent experiments were presented as mean ± standard deviation and analyzed using independent sample *t*-test between two groups and using one-way ANOVA among multiple groups. Tumor size across indicated time points was compared using two-way ANOVA

Given the correlation of PES1 expression to the development of prostate cancer by microarray-based analysis, we aimed to explore the specific effects of PES1 on the development of prostate cancer. The results of RT-qPCR showed that mRNA expression of PES1 was decreased in PC-3 cells with PES1 silencing (Fig. 2a), confirming the knockdown efficiency of PES1 in PC-3 cells. In addition, EdU assay (Fig. 2b), flow cytometry (Fig. 2c), and Transwell assay (Fig. 2d) were respectively conducted to measure cell proliferation, apoptosis, migration and invasion. The results showed that compared with the si-NC-treated cells, si-PES1-treated cells exhibited reduced EdU-positive rate, elevated apoptosis rate, and attenuated cell migration and invasion abilities (all *p* < 0.05). Western blot analysis was performed to measure the protein expression of factors related to cell proliferation (Ki67 and PCNA) (Fig. 2b), apoptosis (c-caspase-3/caspase-3 and c-caspase-9/caspase-9) (Fig. 2c), migration and invasion (MMP-2 and MMP-9) (Fig. 2d). The results revealed that in comparison with the si-NC treatment, si-PES1 treatment resulted in downregulated expression of Ki67, PCNA, MMP-2, and MMP-9 and upregulated expression of c-caspase-3/caspase-3 and c-caspase-9/caspase-9 (all *p* < 0.05). Also, cells transfected with sh-NC or sh-PES1 plasmids were injected into the nude mice to investigate the role of PES1 in prostate cancer in vivo. The results indicated that compared with sh-NC treatment, sh-PES1 treatment caused reduced PES1 mRNA expression as well as decreased tumor growth and tumor volume (*p* < 0.05) (Fig. 2e, f). These results demonstrated that downregulation of PES1 dampened prostate cancer progression.

### miR-1271 specifically binds to PES1 and downregulates its expression

**Fig. 3:**
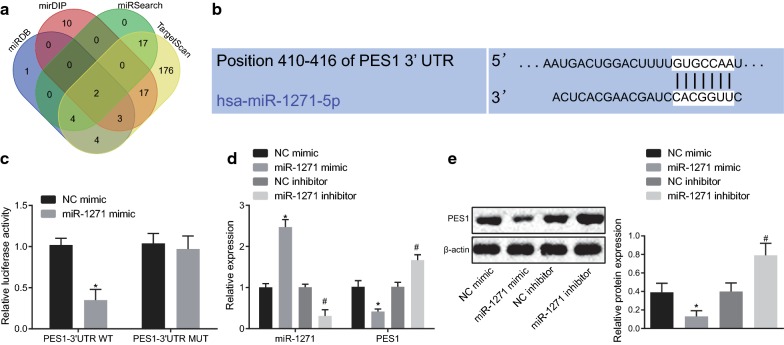
miR-1271 interacts with PES1 and negatively regulates PES1 expression. **a** Regulatory miRNAs of PES1 predicted using the mirDIP, miRDB, miRSearch, and TargetScan databases; four ellipses refer to the prediction results in four databases respectively and the intersection among these ellipses indicating overlapped results. **b** Binding site between miR-1271 and PES1 predicted using the TargetScan. **c** Luciferase activity in response to co-transfection of miR-1271 mimic and NC mimic or PES1-3′UTR-Wt and PES1-3′UTR-Mut, as detected by dual-luciferase reporter gene assay. **d** Expression of miR-1271 and PES1 following treatment with miR-1271 mimic, miR-1271 inhibitor or their respective NCs, as determined using RT-qPCR. **e** Protein expression of PES1 in response to miR-1271 mimic, miR-1271 inhibitor or their respective NCs tested by Western blot analysis. *p < 005 vs. treatment with NC mimic; #*p* < 0.05 vs. treatment with NC inhibitor. Data obtained from three independent experiments were presented as mean ± standard deviation and analyzed using independent sample *t*-test between two groups and using one-way ANOVA among multiple groups

After exploring the effects of PES1 on prostate cancer development, we then focused on the upstream regulatory mechanism of PES1. The mirDIP, miRDB, miRSearch, and TargetScan databases were used to predict the regulatory miRNAs of PES1 and the results indicated that miR-96 and miR-1271 were in the intersection (Fig. 3a).

A specific binding site between PES1 gene sequence and miR-1271 sequence was predicted using the TargetScan (Fig. 3b). Next, dual-luciferase reporter gene assay was conducted to confirm the predicted relationship, the results of which displayed that compared with the co-transfection of NC mimic and PES1-3′UTR-Wt, co-transfection of miR-1271 mimic and PES1-3′UTR-Wt led to reduced luciferase activity (*p* < 0.05). However, there was no significant difference in the luciferase activity between the co-transfection of miR-1271 mimic and PES1-3′UTR-Mut and co-transfection of NC mimic and PES1-3′UTR-Mut (*p* > 0.05) (Fig. 3c), suggesting that miR-1271 can specifically bind to PES1.

Furthermore, the results of RT-qPCR and Western blot analysis illustrated that miR-1271 expression was increased while PES1 expression was decreased following miR-1271 mimic treatment compared with NC mimic treatment (both *p* < 0.05). In contrast to NC inhibitor treatment, miR-1271 inhibitor treatment decreased miR-1271 expression but increased PES1 expression (both *p* < 0.05) (Fig. 3d, e). Collectively, PES1 was a target gene of miR-1271.

### miR-1271 is poorly expressed in prostate cancer tissues and cells

**Fig. 4:**
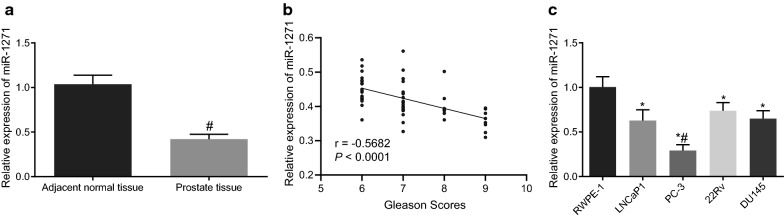
miR-1271 is poorly expressed in prostate cancer tissues and cells. **a** Expression of miR-1271 in prostate cancer and adjacent normal tissues detected by RT-qPCR; #*p* < 0.05 vs. adjacent normal tissues; **b** Correlation between miR-1271 expression and Gleason scoring in prostate cancer tissues analyzed by Pearson’s correlation coefficient. **c** Expression of miR-1271 in prostate cancer cell lines (LNCaP, PC-3, 22Rv1, and DU145) and normal prostatic epithelial cell line (RWPE-1); *p < 0.05 vs. normal prostatic epithelial cell line (RWPE-1); #*p* < 0.05 vs. LNCaP, 22Rv1, and DU145 cell lines. Data obtained from three independent experiments were presented as mean ± standard deviation and analyzed using independent sample *t*-test between two groups and using one-way ANOVA among multiple groups. Pearson’s correlation coefficient was used for analyzing the correlation between miR-1271 expression and Gleason scoring

In order to understand the correlation between miR-1271 expression and prostate cancer, the expression of miR-1271 in prostate cancer tissues and cells was determined using RT-qPCR. As shown in Fig. 4a, the expression of miR-1271 was lower in prostate cancer tissues than that in adjacent normal tissues (*p* < 0.05). Pearson’s correlation coefficient revealed an adverse relationship between miR-1271 expression and Gleason scoring in prostate cancer tissues (Fig. 4b). Moreover, the expression of miR-1271 was also evaluated in prostate cancer cell lines (LNCaP, PC-3, 22Rv1, and DU145) and normal prostatic epithelial cell line (RWPE-1). The results displayed that the expression of miR-1271 was significantly lower in prostate cancer cell lines compared with the normal prostatic epithelial cell lin2e. Particularly, the expression of miR-1271 was the lowest in PC-3 cell lines (*p* < 0.05) (Fig. 4c), and therefore, PC-3 cell lines were selected for subsequent use.

### Elevated miR-1271 suppresses the progression of prostate cancer both in vivo and in vitro

**Fig. 5:**
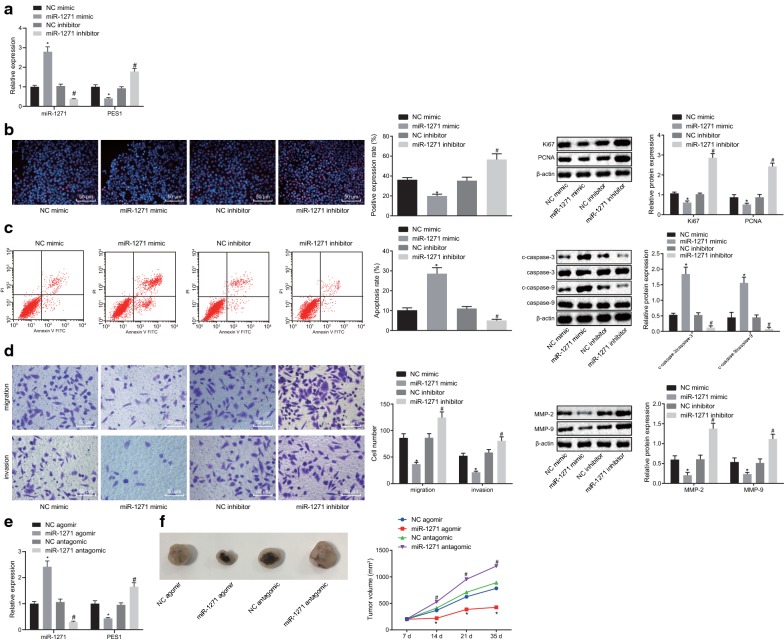
Elevated expression of miR-1271 suppresses the progression of prostate cancer both in vivo and in vitro. **a** miR-1271 expression and PES1 mRNA expression in PC-3 cells detected by RT-qPCR. **b** Images of EdU assay (200×), positive rate of PC-3 cells as well as the expression of Ki67 and PCNA, following treatment of NC mimic, miR-1271 mimic or their respective NC detected by EdU assay and Western blot analysis. **c** PC-3 cell apoptosis and protein expression of c-caspase-3/caspase-3 and c-caspase-9/caspase-9 in PC-3 cells following treatment of NC mimic, miR-1271 mimic or their respective NC tested by flow cytometry and Western blot analysis. **d** Images (200 ×) and quantitative analysis of PC-3 cell migration and invasion and protein expression of MMP-2 and MMP-9 in PC-3 cells following treatment of NC mimic, miR-1271 mimic or their respective NC evaluated by Transwell assay and Western blot analysis. **e** miR-1271 expression and PES1 mRNA expression in mouse tumors detected by RT-qPCR. **f** Representative tumors and tumor growth rate of mice bearing human prostate cancer cell xenografts in response to NC agomir, miR-1271 antagomir or their respective NC (n = 6 for each group). *p < 0.05, vs. treatment with NC mimic or NC agomir. #*p* < 0.05, vs. treatment with NC inhibitor or NC antagomir. Data obtained from three independent experiments were presented as mean ± standard deviation and analyzed using independent sample *t*-test between two groups and using one-way ANOVA among multiple groups. Tumor size over indicated time points were compared using two-way ANOVA

On the basis that downregulation of miR-1271 was observed in prostate cancer, the PC-3 cells were transfected with plasmids of miR-1271 mimic, miR-1271 inhibitor or their respective NCs to reveal the effects of miR-1271 on the development of prostate cancer. Initially, RT-qPCR was conducted to detect the expression of miR-1271 and PES1, the results of which showed an enhancement in miR-1271 expression and a decline in PES1 mRNA expression in the presence of miR-1271 mimic. However, miR-1271 downregulation decreased miR-1271 expression while elevating PES1 mRNA expression (Fig. 5a). EdU assay (Fig. 5b), flow cytometry (Fig. 5c), and Transwell assay (Fig. 5d) were performed to evaluate the effects of miR-1271 on cell proliferation, apoptosis, migration, and invasion in vitro. We found that relative to PC-3 cells transfected with NC mimic plasmids, PC-3 cells transfected with miR-1271 mimic plasmids had decreased positive rate, increased apoptosis rate, reduced migration and invasion abilities (all *p* < 0.05), which was opposite to the results found in PC-3 cells transfected with miR-1271 inhibitor plasmids comparing to its control counterpart (NC inhibitor) (all *p* < 0.05). Additionally, expression of factors related to proliferation (Ki67 and PCNA) (Fig. 5b), apoptosis (c-caspase-3/caspase-3 and c-caspase-9/caspase-9) (Fig. 5c), and migration and invasion (MMP-2 and MMP-9) (Fig. 5d) was tested by Western blot analysis. Our data showed that in comparison with NC mimic plasmid, miR-1271 mimic plasmid transfection downregulated expression of Ki67, PCNA, MMP-2, and MMP-9, but upregulated expression of c-caspase-3/caspase-3 and c-caspase-9/caspase-9 (all *p* < 0.05). Further, the nude mice were inoculated with miR-1271 agomir- or miR-1271 antagomir-treated cells to assess the effects of miR-1271 on tumor growth in vivo. The results indicated that compared with NC agomir treatment, tumor growth rate and tumor volume were decreased while miR-1271 expression was upregulated in response to miR-1271 agomir treatment (*p* < 0.05). In contrast to NC antagomir delivery, miR-1271 antagomir delivery caused reduced miR-1271 expression along with increased tumor growth rate and tumor volume (*p* < 0.05) (Fig. 5e, f). Based on the above findings, it was suggested that miR-1271 could attenuate cell proliferation, migration, and invasion, as well as tumor growth, but potentiated cell apoptosis in prostate cancer.

### Elevated miR-1271 suppresses prostate cancer development by reducing the expression of PES1

**Fig. 6:**
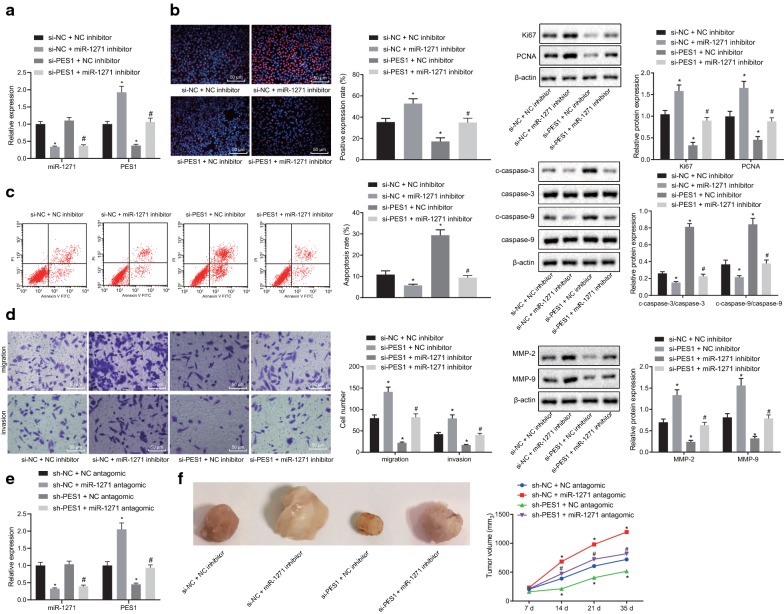
miR-1271 negatively regulates PES1 to impede the development of prostate cancer. PC-3 cells were transfected with sh-PES1 coupled with NC inhibitor or both sh-PESE1 coupled with miR-1271 inhibitor. **a** miR-1271 expression and PES1 mRNA expression in PC-3 cells detected by RT-qPCR. **b** Images of EdU assay (200×), positive rate of PC-3 cells as well as the expression of Ki67 and PCNA in PC-3 cells detected by EdU assay and Western blot analysis. **c** Cell apoptosis and protein expression of c-caspase-3/caspase-3 and c-caspase-9/caspase-9 in PC-3 cells tested by flow cytometry and Western blot analysis. **d** Images (200 ×) and quantitative analysis of cell migration and invasion and protein expression of MMP-2 and MMP-9 in PC-3 cells evaluated by Transwell assay and Western blot analysis. **e** miR-1271 expression and PES1 mRNA expression in mouse tumors detected by RT-qPCR. **f** Representative tumors and tumor growth rate of mice bearing human prostate cancer cell xenografts in response to co-treatment of sh-PES1 and NC antagomir or co-treatment of sh-PES1 and miR-1271 antagomir (n = 6 for each group). *p < 0.05, vs. co-treatment of sh-NC and NC inhibitor or co-treatment of sh-NC and NC antagomir; #*p* < 0.05, vs. co-treatment of sh-PES1 and NC inhibitor or co-treatment of sh-PES1 and NC antagomir. Data obtained from three independent experiments were presented as mean ± standard deviation and tested using one-way ANOVA among multiple groups. Tumor size over indicated time points was compared using two-way ANOVA

According to our data described above, both miR-1271 and PES1 played an essential role in the development of prostate cancer. We then further elucidated whether miR-1271 participates in prostate cancer progression by regulating PES1. RT-qPCR was conducted to detect the expression of miR-1271 and PES1, and the results of which showed a decline in PES1 mRNA expression in response to PES1 silencing yet an increase in response to miR-1271 knockdown. In addition, PES1 mRNA expression was upregulated in PC-3 cells transfected with both sh-PES1 and miR-1271 inhibitor plasmids (Fig. 6a). As detected by EdU assay (Fig. 6b), flow cytometry (Fig. 6c), and Transwell assay (Fig. 6d), respectively, PC-3 cells transfected with both sh-PES1 and NC inhibitor plasmids presented reduced positive rate (*p* < 0.05), enhanced apoptosis rate (*p* < 0.05), diminished migration and invasion abilities (*p* < 0.05) compared with those cells manipulated with both sh-NC and NC inhibitor plasmids, which was abrogated by dual transfection with sh-NC and miR-1271 inhibitor plasmids. On the contrary, PC-3 cells co-treated with sh-PES1 and miR-1271 inhibitor plasmids exhibited a negative correlation while comparing with the PC-3 cells co-treated with sh-PES1 and NC inhibitor plasmids (*p* < 0.05). In addition, the results of Western blot analysis implied that the expression of proliferation-related factors (Ki67 and PCNA) (Fig. 6b) and migration- and invasion-related factors (MMP-2 and MMP-9) (Fig. 6d) was downregulated while that of apoptosis-related factors (c-caspase-3/caspase-3 and c-caspase-9/caspase-9) (Fig. 6c) was upregulated in PC-3 cells co-transfected with sh-PES1 and NC inhibitor plasmids relative to the co-treatment of sh-NC and NC inhibitor plasmids. An opposite trend was observed in PC-3 cells co-transfected with sh-NC and miR-1271 inhibitor or sh-PES1 and NC inhibitor plasmids (all *p* < 0.05). Moreover, in comparison with the mice inoculated with cells co-treated with sh-NC and NC antagomir, mice inoculated with cells co-treated with sh-PES1 and NC antagomir had decreased PES1 mRNA expression and reduced tumor growth rate and tumor volume, which was negated by co-treatment with sh-NC and miR-1271 antagomir (both *p* < 0.05). Relative to co-treatment of sh-PES1 and NC antagomir, dual treatment with sh-PES1 and miR-1271 antagomir resulted in augmented PES1 mRNA expression along with elevated tumor growth rate and tumor volume (both *p* < 0.05) (Fig. 6e, f). Taken together, these results indicated that miR-1271 could downregulate PES1 to inhibit prostate cancer cell proliferation, migration, and invasion along with tumor growth and simultaneously, potentiate cell apoptosis.

### miR-1271 activates the ERβ signaling pathway in prostate cancer cells

**Fig. 7:**
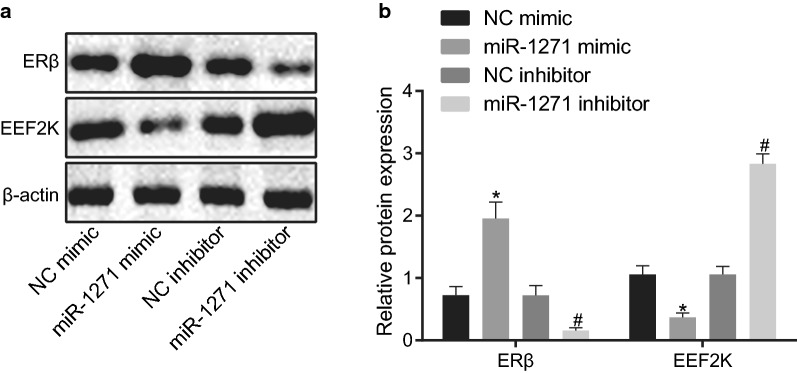
The ERβ signaling pathway is activated by the elevation of miR-1271 in prostate cancer cells. **a**, **b**, Protein bands (**a**) and quantitative analysis (**b**) of the expression of ERβ, EEF2K, and MNK1/2 in response to overexpression or depletion of miR-1271, detected by Western blot analysis. *p < 0.05 vs. NC mimic treatment; *p < 0.05 vs. NC inhibitor treatment. Data obtained from three independent experiments were presented as mean ± standard deviation and tested using one-way ANOVA among multiple groups

Next, we aimed to explore the effects of miR-1271 on its downstream signaling pathway. Western blot analysis was conducted to detect the protein expression of the ERβ signaling pathway-related factors in PC-3 cells. The results revealed that compared with NC mimic treatment, miR-1271 mimic treatment contributed to elevated expression of ERβ (*p* < 0.05), but reduced expression of EEF2K and MNK1/2 (*p* < 0.05). These data were opposite to the trend found in PC-3 cells treated with miR-1271 inhibitor versus the PC-3 cells treated with NC inhibitor (*p* < 0.05) (Fig. 7a, b). In summary, miR-1271 elevation activated the ERβ signaling pathway.

### Silencing of PES1 inhibits the progression of prostate cancer in vivo and in vitro by activating the ERβ signaling pathway

**Fig. 8:**
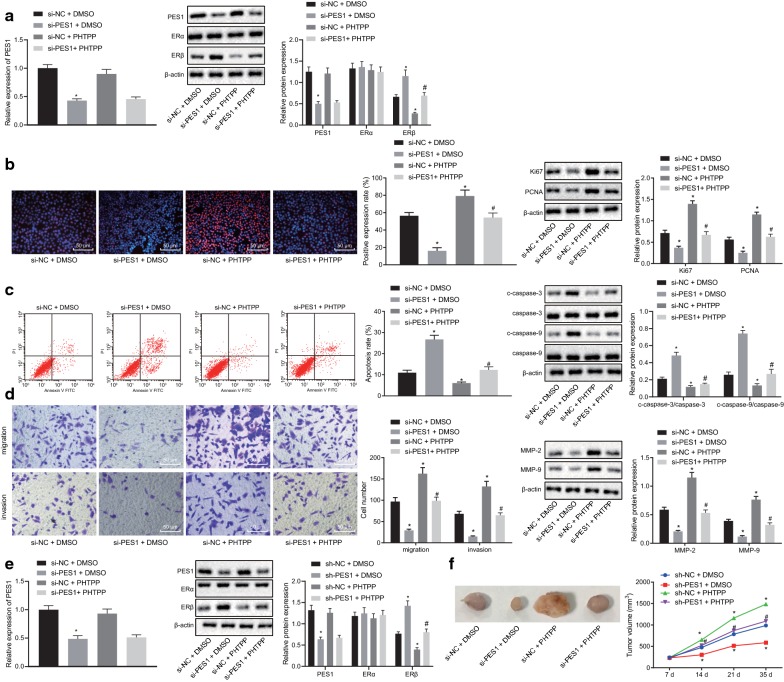
Silencing of PES1 inhibits the progression of prostate cancer in vivo and in vitro by activating the ERβ signaling pathway. The cells were transfected with both si-PES1 and DMSO or both si-PES1 and PHTPP. **a** mRNA and protein expression of PES1, ERα and ERβ in PC-3 cells detected by RT-qPCR and Western blot analysis. **b** Images of EdU assay (200×), positive rate of cells and the expression of Ki67 and PCNA in PC-3 cells determined using EdU assay and Western blot analysis. **c** PC-3 cell apoptosis and protein expression of c-caspase-3/caspase-3 and c-caspase-9/caspase-9 in PC-3 cells tested by flow cytometry and Western blot analysis. **d** Images (200×) and quantitative analysis of cell migration and invasion and protein expression of MMP-2 and MMP-9 in PC-3 cells evaluated by Transwell assay and Western blot analysis. **e** mRNA and protein expression of PES1, ERα and ERβ in mouse tumors detected by RT-qPCR and Western blot analysis. **f** Representative tumors and tumor growth rate of mice bearing human prostate cancer cell xenografts in response to co-treatment of sh-PES1 and DMSO or co-treatment of sh-PES1 and PHTPP (n = 6 for each group). *p < 0.05, vs. co-treatment of sh-NC and DMSO or co-treatment of sh-NC and DMSO; #*p* < 0.05, vs. co-treatment of sh-PES1 and DMSO or co-treatment of sh-PES1 and DMSO. Data obtained from three independent experiments were presented as mean ± standard deviation and tested using one-way ANOVA among multiple groups. Tumor size over indicated time points was compared using two-way ANOVA

We then examined the role of PES1 in prostate cancer progression via the ERβ signaling pathway. RT-qPCR and Western blot analysis were conducted to detect the mRNA and protein expression of PES1, ERα and ERβ. The results revealed that PES1 could control ERα expression but not ERβ expression (Fig. 8a). EdU assay (Fig. 8b), flow cytometry (Fig. 8c), and Transwell assay (Fig. 8d) were conducted to detect cell proliferation, apoptosis, migration and invasion, respectively and Western blot analysis was conducted to measure the protein expression of factors related to cell proliferation (Ki67 and PCNA), apoptosis (c-caspase-3/caspase-3 and c-caspase-9/caspase-9), migration and invasion (MMP-2 and MMP-9). Our results revealed that compared with the cells treated with sh-NC, cells treated with sh-PES1 displayed reduced positive rate, attenuated migration and invasion abilities, and increased apoptosis rate, accompanied by downregulated Ki67, PCNA, MMP-2, and MMP-9 and upregulated c-caspase-3/caspase-3 and c-caspase-9/caspase-9, which was undermined following co-treatment of sh-NC and PHTPP (all *p* < 0.05). However, co-treatment of sh-PES1 and PHTPP resulted in an opposite trend relative to the co-treatment of si-PES1 and DMSO (all *p* < 0.05). Moreover, the results from in vivo study revealed that in contrast to the co-treatment of sh-NC, treatment of sh-PES1 resulted in elevated PES1 expression and decreased tumor growth rate and tumor volume, while co-treatment of sh-NC and PHTPP downregulated ERβ expression and increased tumor growth rate and tumor volume (*p* < 0.05). Compared with the co-treatment of sh-PES1 and DMSO, co-treatment of sh-PES1 and PHTPP led to diminished ERβ expression and increased tumor growth rate and tumor volume (*p* < 0.05) (Fig. 8e, f), indicating PES1 deactivated ERβ in prostate cancer (Additional file 2).

## Discussion

Prostate cancer is a serious disease featured with high fatality and morbidity rates [18]. However, effective treatment to this disease still faces a big challenge due to the various clinical behaviors [19]. As indicated by microarray-based analysis, PES1 was screened as a DEG associated with the progression of prostate cancer and was a downstream mRNA of miR-1271. Therefore, this study aimed to investigate the underlying mechanism of PES1 and miR-1271 in modulating the development of prostate cancer. Our findings provide evidence that miR-1271 downregulated PES1 to activate the ERβ signaling pathway, which further inhibited the biological processes of prostate cancer.

Initially, we found that PES1 was highly expressed while miR-1271 was marginally expressed in prostate cancer. Consistently, Li et al. [20] found that PES was abundantly expressed in many cancer cells, including colon cancer, breast cancer, and ovarian cancer. Among these studies, PES was indicated to be involved in the progression of cancer and acted as a diagnostic and therapeutic biomarker for cancers. Likewise, colon cancer tissues displayed elevated expression of PES1, and in the absence of PES1, attenuated proliferation and tumor growth of colon cancer cell was observed, suggesting that downregulated PES1 functions as a tumor suppressor [21]. Moreover, miRNAs were found to exert promotive or inhibitory effects on prostate cancer and many miRNAs, including miR-221 and miR-205 were reported to be poorly expressed in prostate cancer tissues [22]. Additionally, miR-1271 expression was downregulated in prostate cancer tissues and its elevation remarkably hindered the progression of prostate cancer by negatively regulating the expression of its target gene DIX domain containing 1 (DIXDC1) [23].

Our data revealed that PES1 was a novel target gene of miR-1271 and the inhibition of PES1 caused by miR-1271 activated the ERβ signaling pathway. miR-1271 was shown to specifically target ETS-related gene (ERG) to further downregulate the expression of ERG and inhibit cellular processes in prostate cancer [11], which was in line with our results. Similarly, elevated miR-1271 worked as an inhibitor in ovarian cancer due to its inhibitory role in cell proliferation and tumor growth by directly targeting cyclin G1 [24]. Furthermore, PES1 expression is found to be negatively associated with ERβ while positively correlated with ERα in breast cancer [12, 25]. Therefore, downregulated PES1 resulted in a delay in the development of ovarian cancer via activated ERβ but attenuated ERα, which further proposed PES1 as a therapeutic target for treatment of ovarian cancer [26], which was consistent with our findings.

More importantly, overexpression of miR-1271 or silencing of PES1 suppressed prostate cancer cell proliferation, migration, and invasion as well as tumor growth, but promoted cell apoptosis. Previously, Liu et al. [10], have shown that overexpression of miR-1271 inhibits cell proliferation, invasion, migration, and tumor metastasis by binding to zinc-finger Ebox binding homeobox 1 (ZEB1) and twist family bHLH transcription factor 1 (TWIST1) in pancreatic cancer, highlighting that miR-1271 can function as a marker for treatment and prognosis of metastatic pancreatic cancer. Also, miR-1271 targeted and downregulated forkhead box protein Q1 to further repress gastric cancer cell invasion, proliferation, and epithelial-mesenchymal transition [27]. In non-small cell lung cancer, upregulation of miR-1271 contributes to the inhibition of cancer development both in vitro and in vivo by downregulation the expression of its target gene mechanistic target of rapamycin (mTOR) [28]. As for PES, Li et al. [29] demonstrated that depletion of PES1 suppressed cell proliferation and attenuated tumorigenicity in breast cancer, suggesting that PES1 could work as a marker for the breast cancer therapy. Moreover, downregulation of PES1 has the potency to facilitate cell apoptosis and attenuate tumor growth in neuroblastoma, thus also acting as a marker to predict the treatment outcome of neuroblastoma [30]. However, these studies did not find upstream regulatory miRNA to mediate the cellular process.

## Conclusions

Taken together, our results demonstrated that overexpression of miR-1271 could potentially repress the progression of prostate cancer via activation of the ERβ signaling pathway by downregulating PES1. This observation highlighted upregulated miR-1271 and downregulated PES1 can be therapeutic predictive markers for prostate cancer. However, more efforts are necessary to elucidate the clinical effects of miR-1271 and PES1 in prostate cancer and relevant diseases in the future studies.

## Supplementary information

**Additional file 1: Table S1.** Transfection sequence.

**Additional file 2.** Original images of Western blot.

## Data Availability

All data generated or analyzed during this study are available.

## Abbreviations

PES1: Pescadillo homolog 1
DEGs: Differentially expressed genes
miRNAs: microRNAs
miR-1271: microRNA-1271
ER: Estrogen receptor
TNM: Tumor-nodes-metastasis
Wt: Wild type
Mut: Mutant
FBS: Fetal bovine serum
NC: Negative control
DMSO: Dimethyl sulfoxide
RT-qPCR: Reverse transcription quantitative polymerase chain reaction
GAPDH: Glyceraldehyde-3-phosphate dehydrogenase
PHTPP: Pyrazolo [1,5-a] pyrimidine
PCNA: Proliferating cell nuclear antigen
TCGA: The Cancer Genome Atlas
ERG: ETS-related gene. 3′-UTR: 3′-untranslated region
3′-UTR: 3′-untranslated region
BSA: Bovine serum albumin
PBS: Phosphate-buffered saline
sh: Short hairpin RNA
DMEM: Dulbecco’s modified Eagle’s medium
EdU: 5-ethynyl-2′-deoxyuridine
ANOVA: Analysis of variance
GEO: Gene Expression Omnibus
DIXDC1: DIX domain containing 1
ERG: ETS-related gene
ZEB1: Zinc-finger Ebox binding homeobox 1
TWIST1: Twist family bHLH transcription factor 1
mTOR: Mechanistic target of rapamycin
