# Supplementary material for: microRNA-1271 impedes the development of prostate cancer by downregulating PES1 and upregulating ERβ
Source: J Transl Med. 2020 May 24;18:209. doi: 10.1186/s12967-020-02349-1 (PMC7245853; doi:10.1186/s12967-020-02349-1)
Supplement: Supplementary file 1 — Additional file 1: Table S1. Transfection sequence. [file 12967_2020_2349_MOESM1_ESM.docx]

**Table S1** Transfection sequence

| Item | Sequence (5’-3’) |
| --- | --- |
| NC mimic | AGTTTCCAATCGTATTAACGCTC |
| miR-1271 mimic | ACTCACGAACGATCCACGGTTC |
| NC inhibitor | GTAGGGCCTTAGATTCCGTAT |
| miR-1271 inhibitor | GAACCGTGGATCGTTCGTGT |
| sh-NC | TTCTCCGAACGTGTCACGT |
| sh-PES1 | ATCATCAAGGAACGGTAT |

Note: NC, negative control; sh, short hairpin RNA; PES1, pescadillo homolog 1.
