# Supplementary material for: microRNA-1271 impedes the development of prostate cancer by downregulating PES1 and upregulating ERβ
Source: J Transl Med. 2020 May 24;18:209. doi: 10.1186/s12967-020-02349-1 (PMC7245853; doi:10.1186/s12967-020-02349-1)
Supplement: Supplementary file 2 — Additional file 2. Original images of Western blot. [file 12967_2020_2349_MOESM2_ESM.docx]

Original Figure 2A


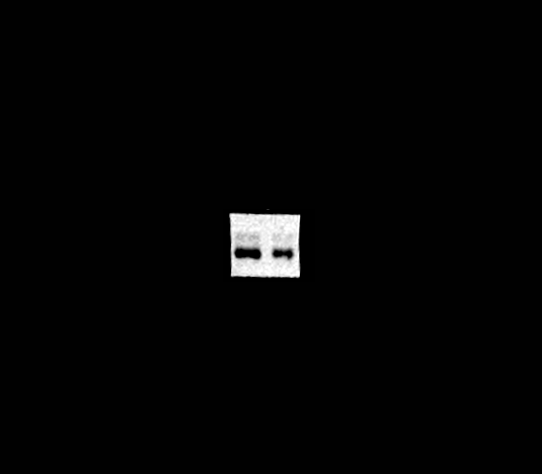


KI67


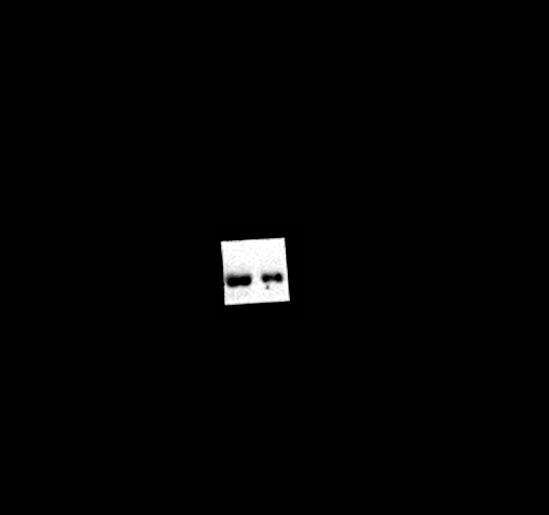


PCNA


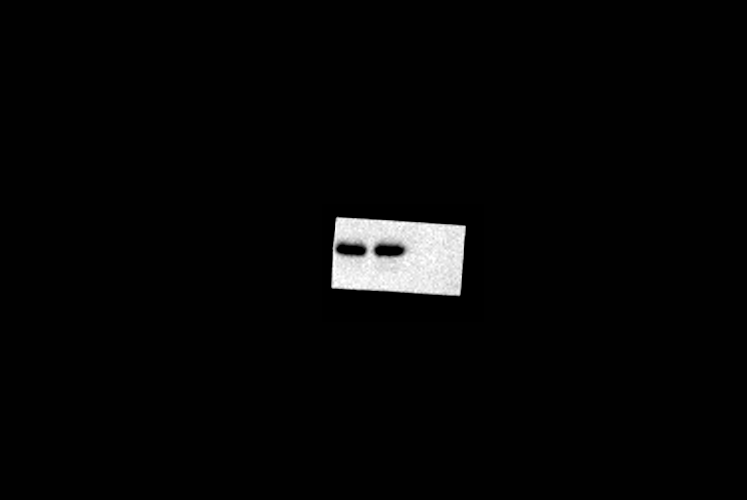


β-actin

Original Figure 2B


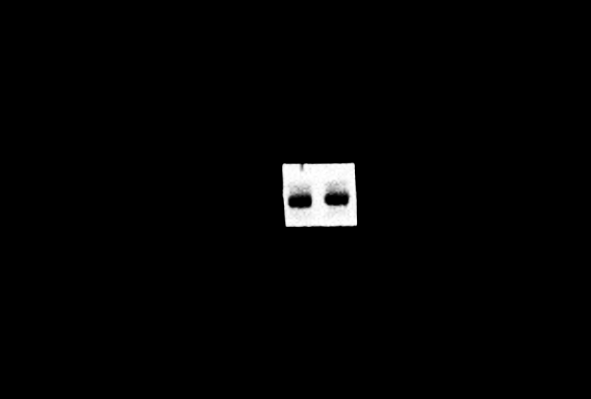


caspase-3


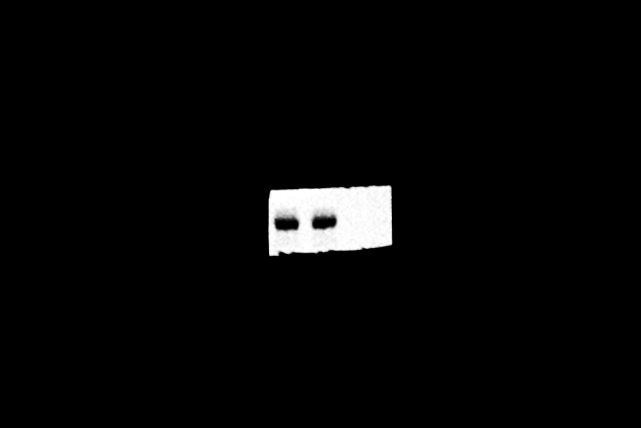


caspase-9


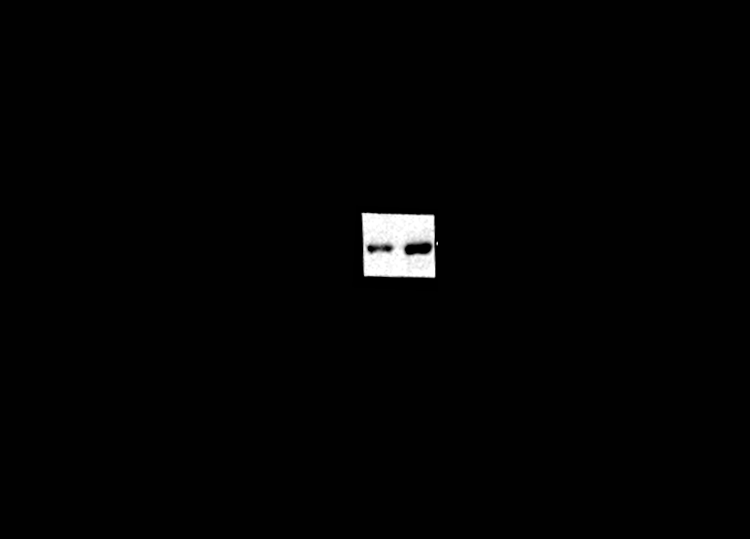


c-caspase-3


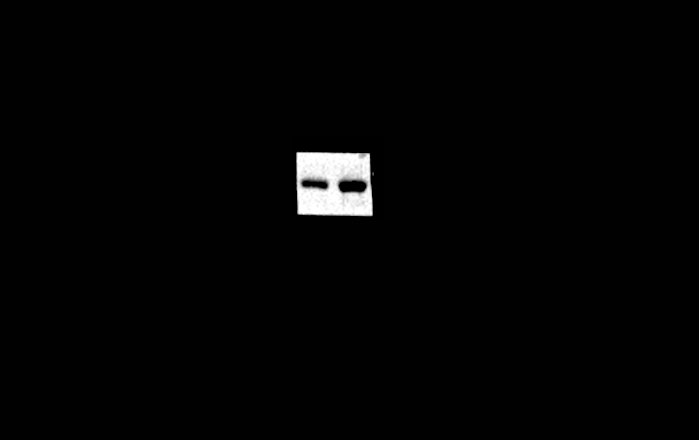


c-caspase-9


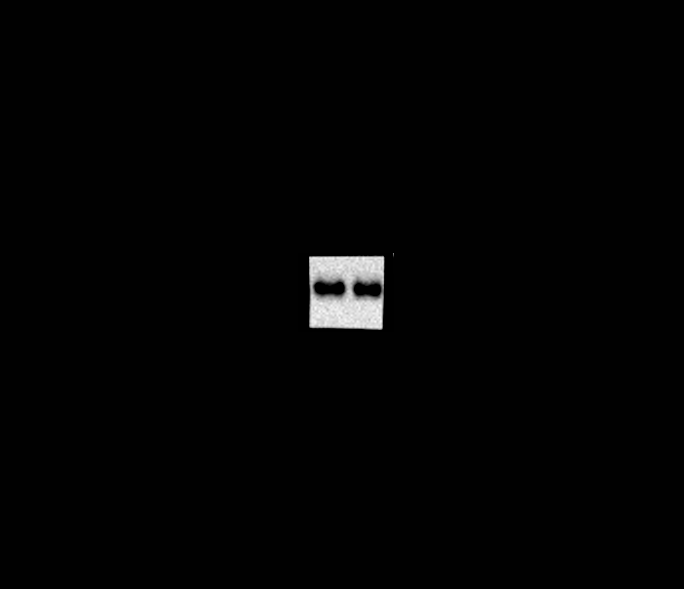


β-actin

Original Figure 2C


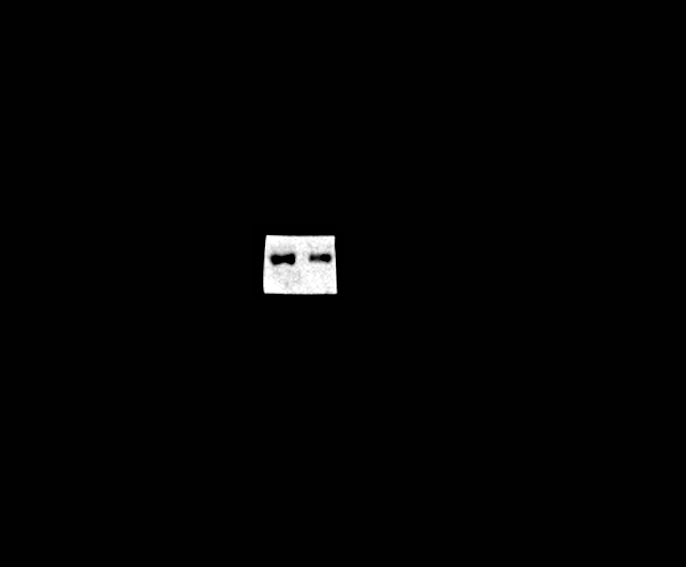


MMP-2


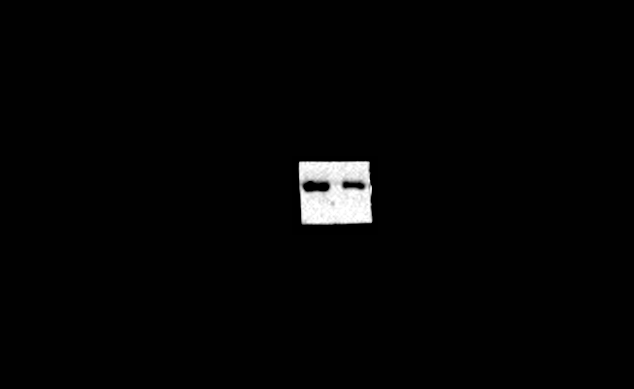


MMP-9


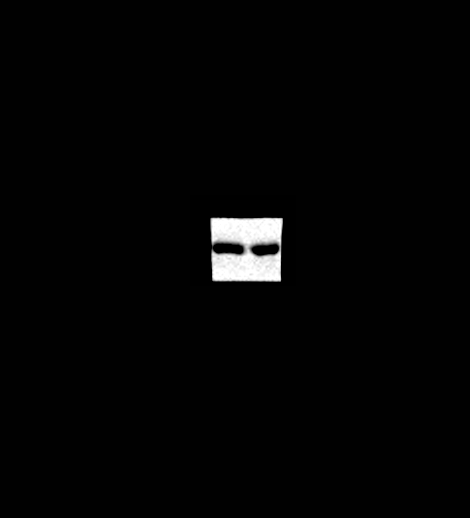


β-actin

Original Figure 3E


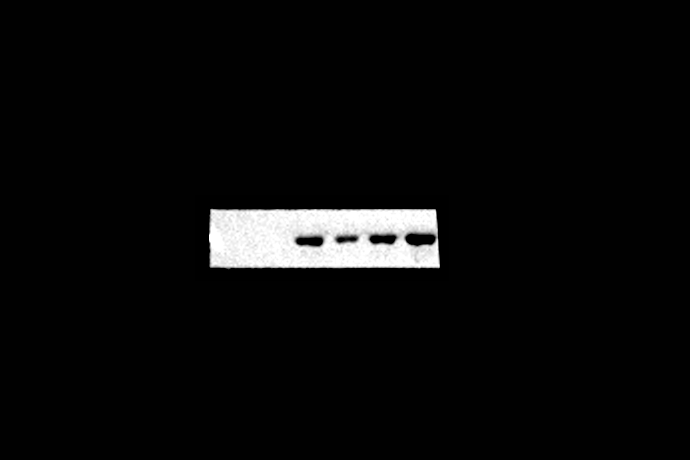


PES1


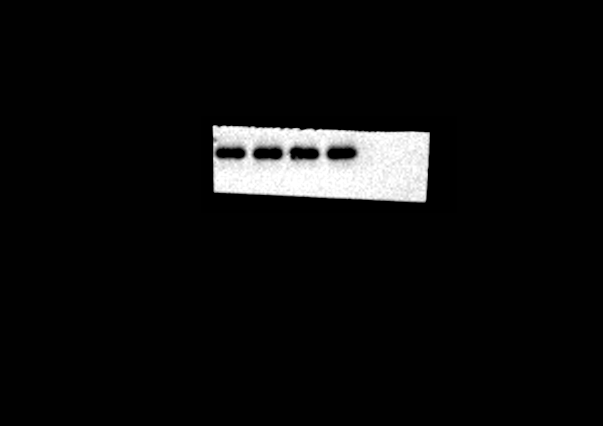


β-actin

Original Figure 5A


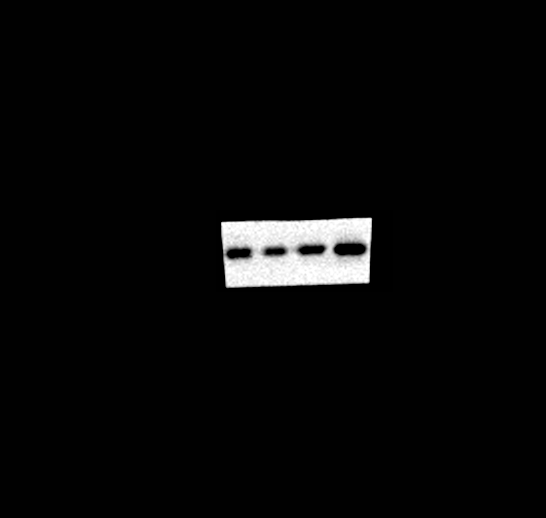


Ki67


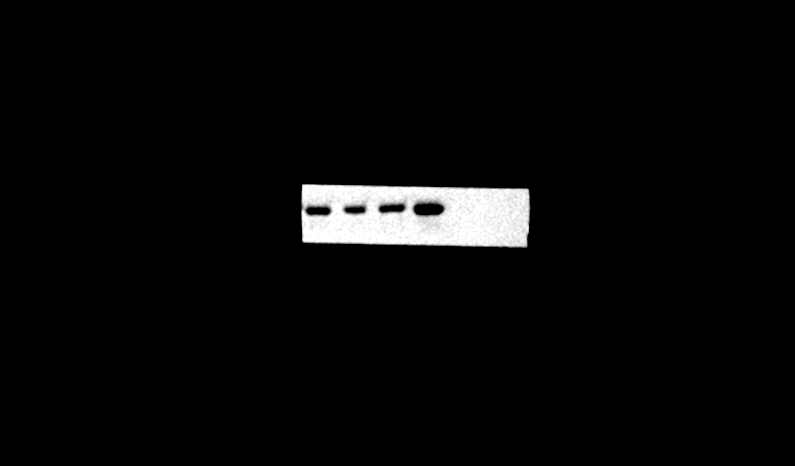


PCNA


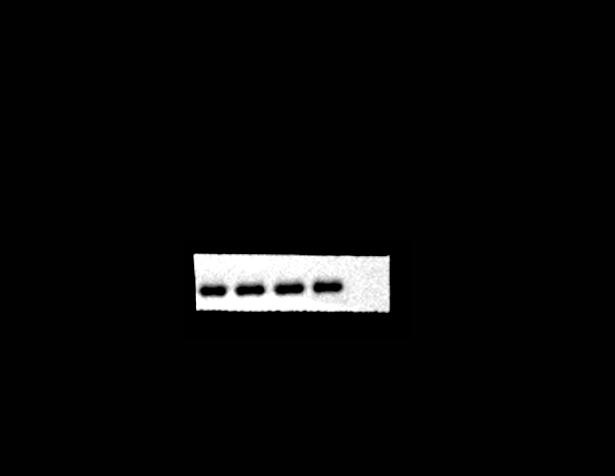


β-actin

Original Figure 5B


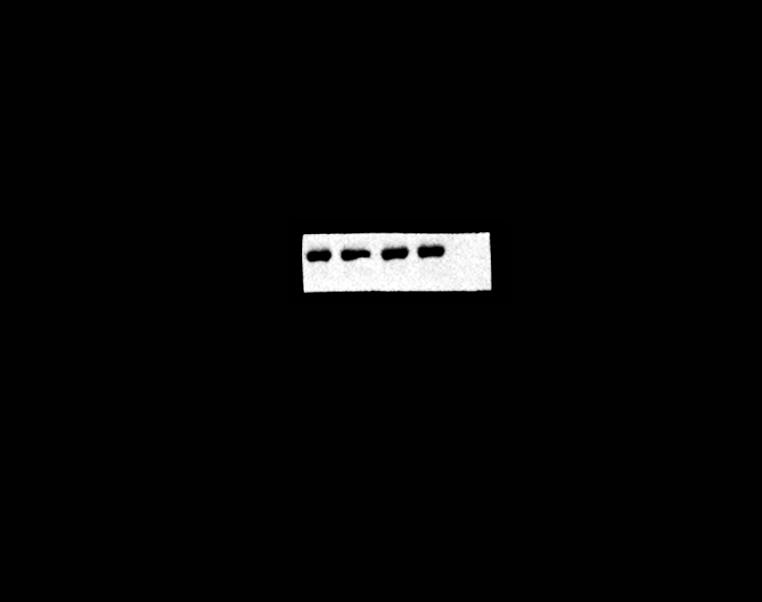


caspase-3


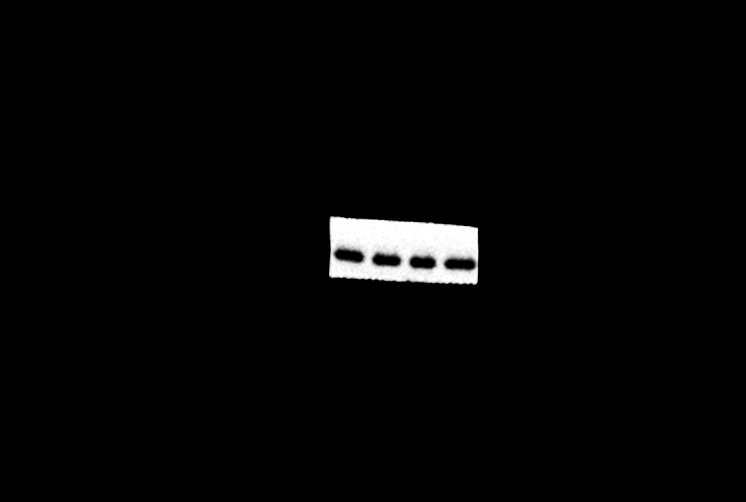


caspase-9


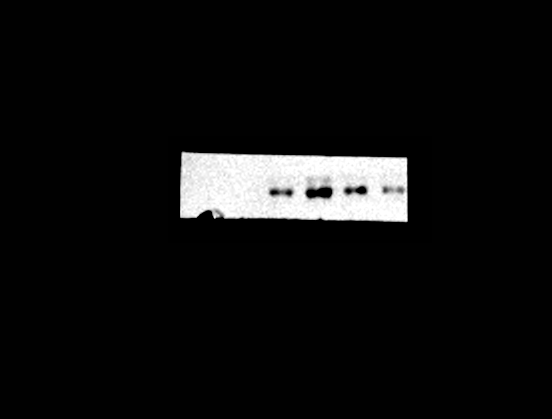


c-caspase-3


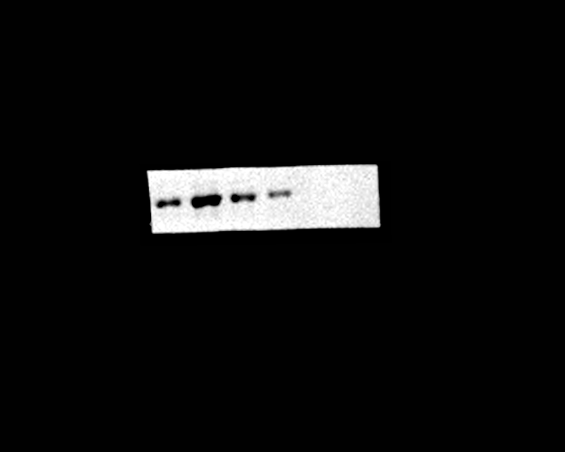


c-caspase-9


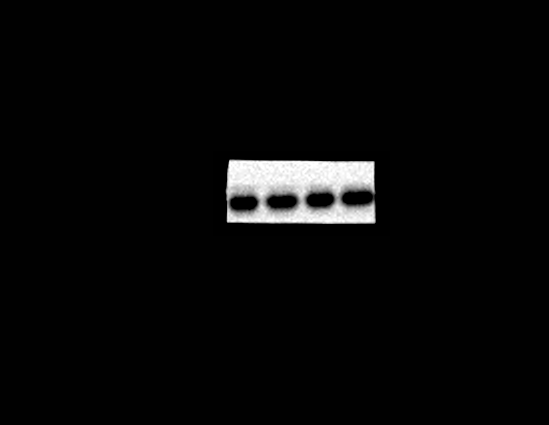


β-actin

Original Figure 5C


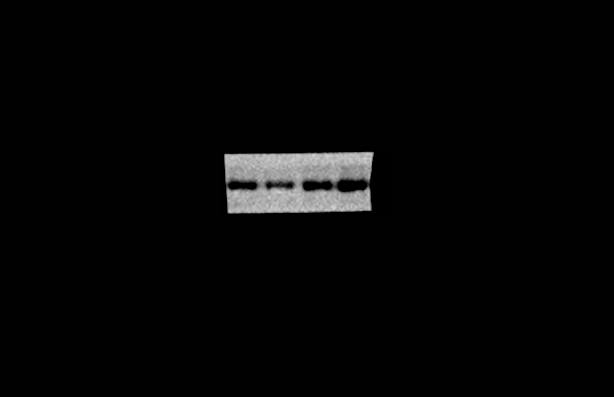


MMP-2


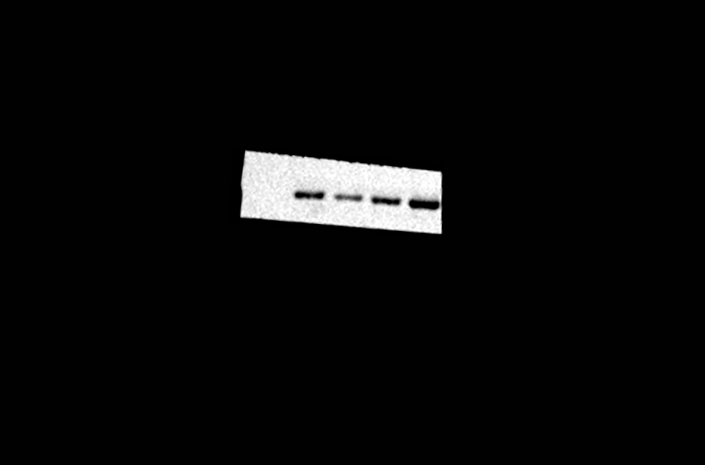


MMP-9


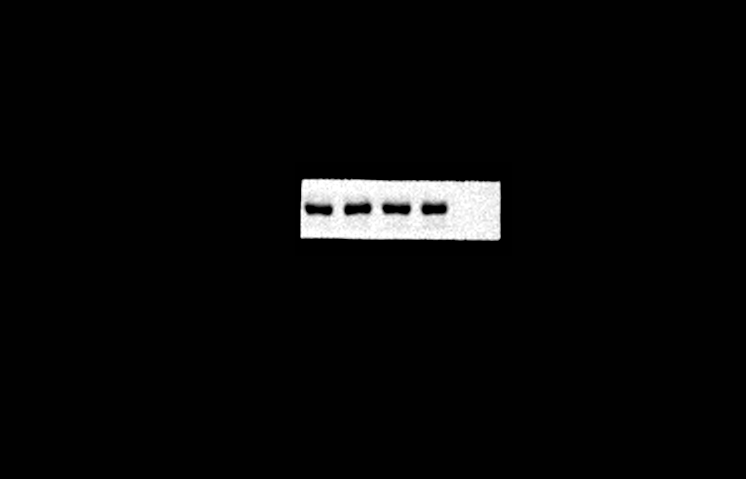


β-actin

Original Figure 6B


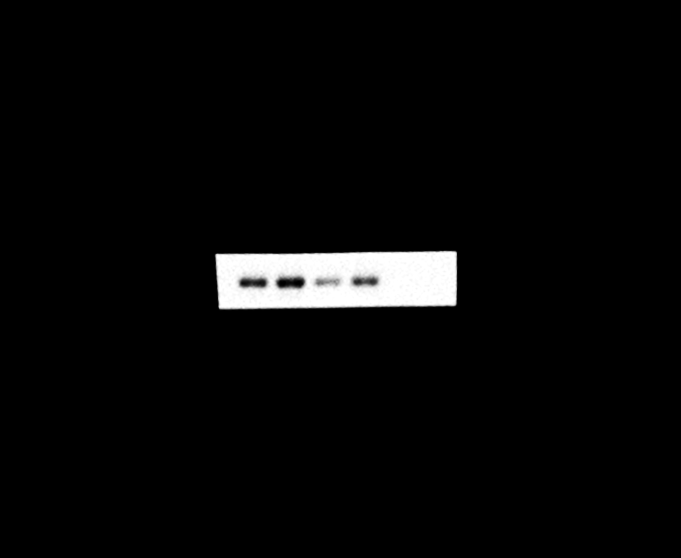


Ki67


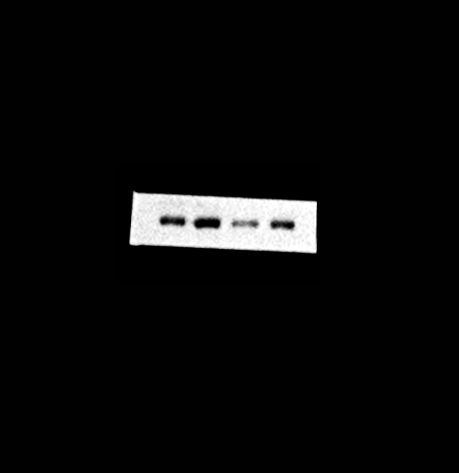


PCNA


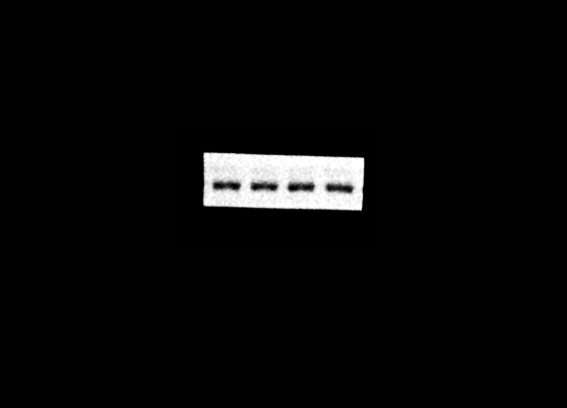


β-actin

Original Figure 6C


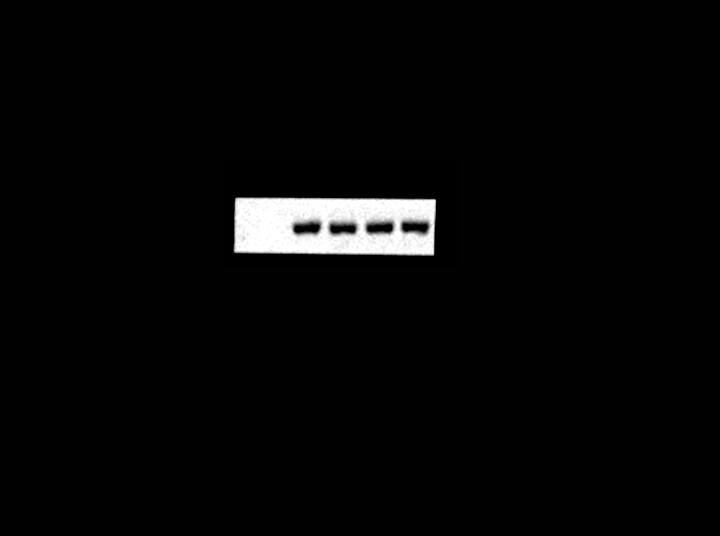


caspase-3


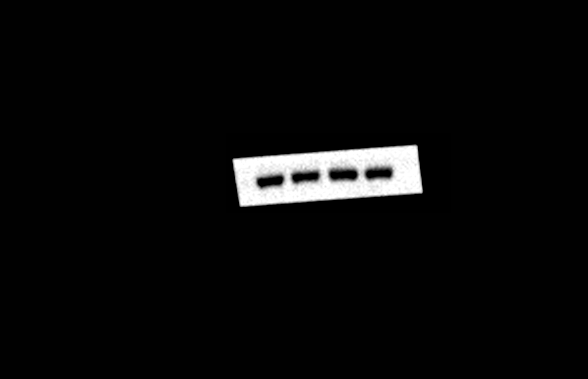


caspase-9


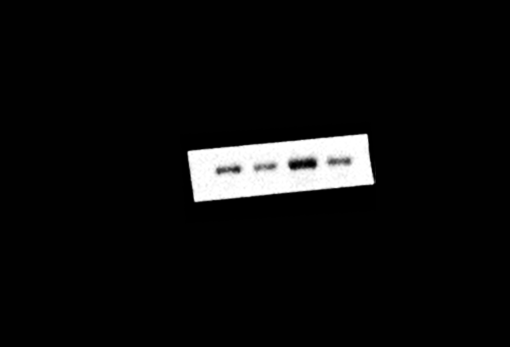


c-caspase-3


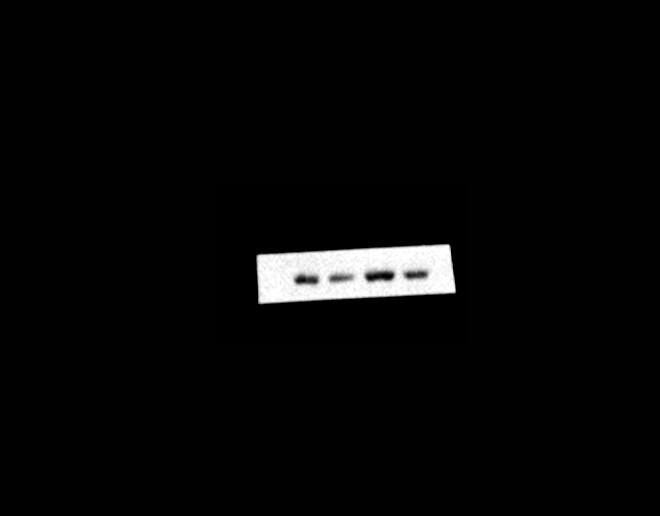


c-caspase-9


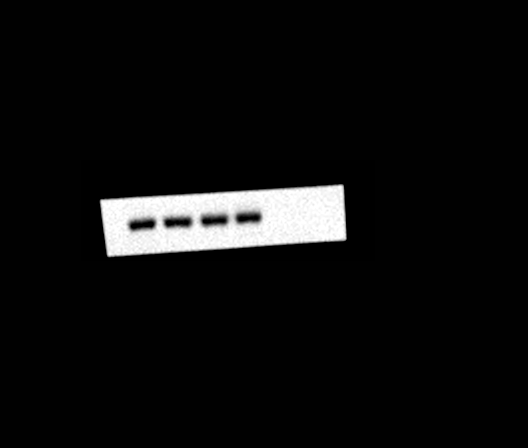


β-actin

Original Figure 6D


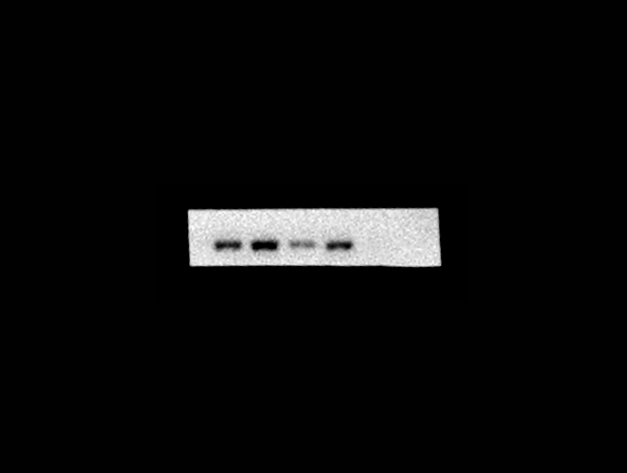


MMP-2


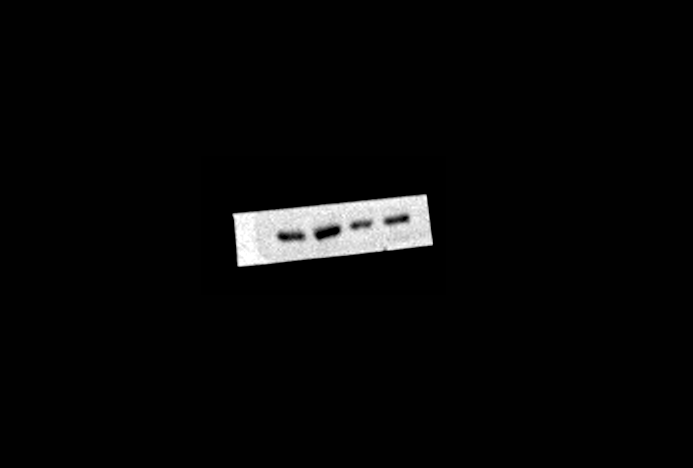


MMP-9


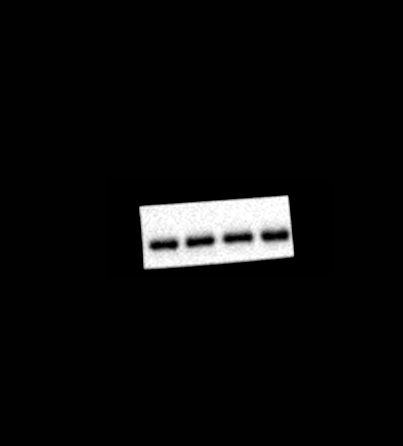


β-actin

Original Figure 7A


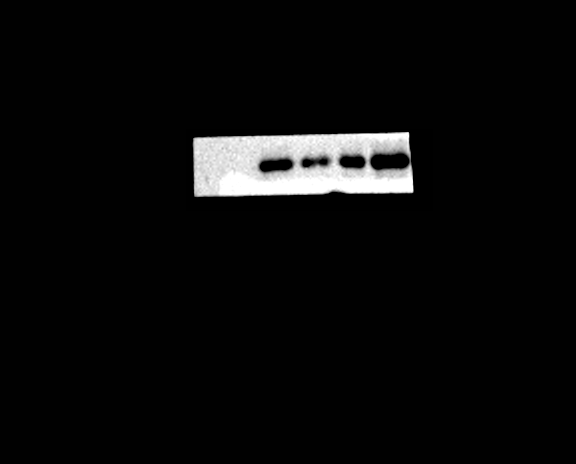


EEF2K


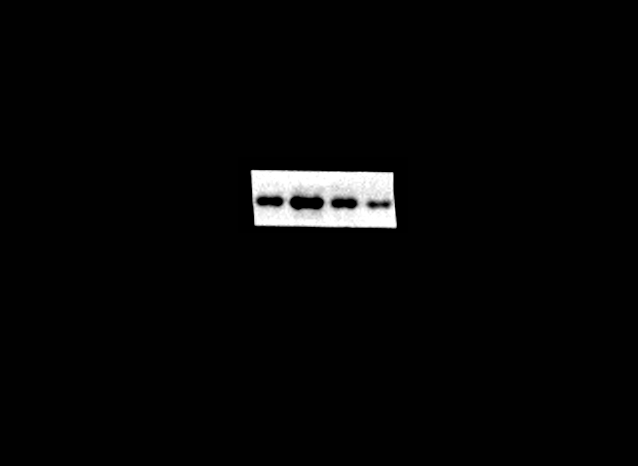


ERβ


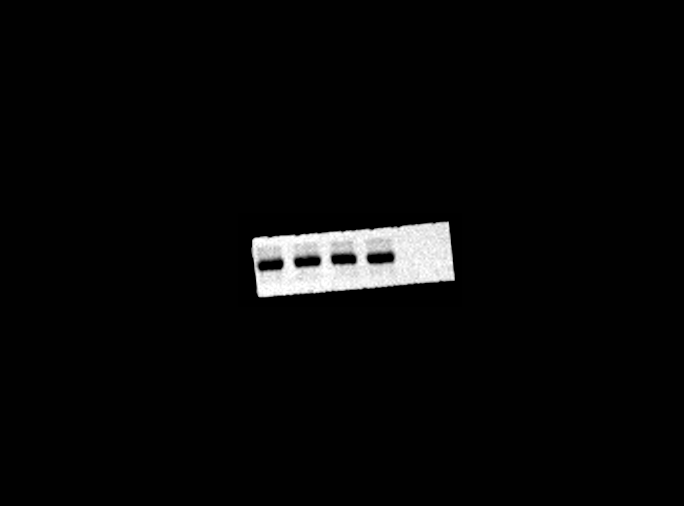


β-actin

Original Figure 8A


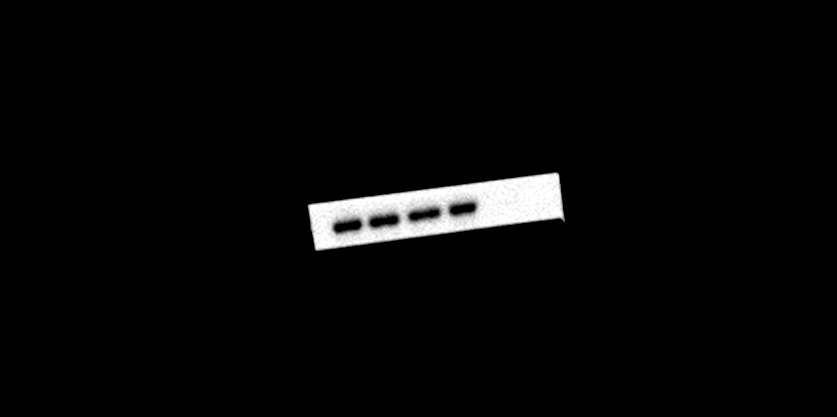


ERα


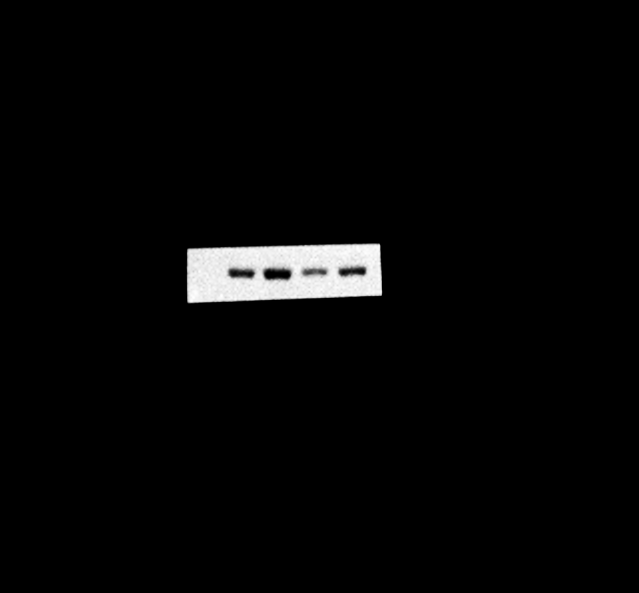


ERβ


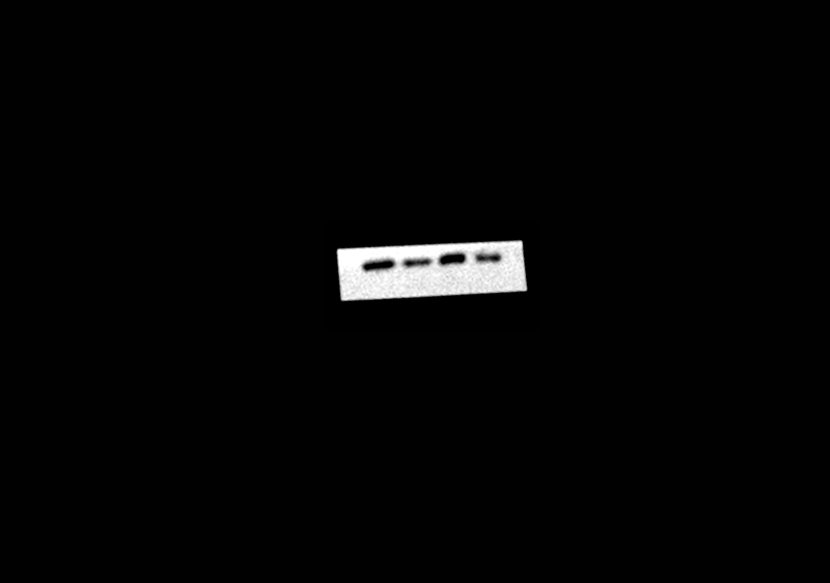


PES1


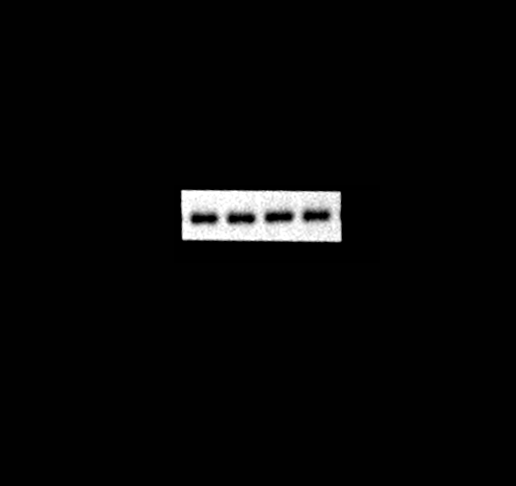


β-actin

Original Figure 8B


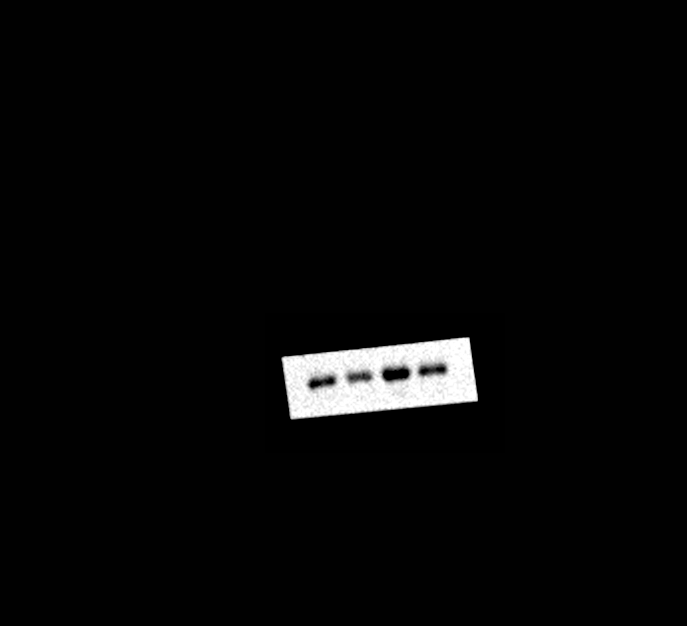


Ki67


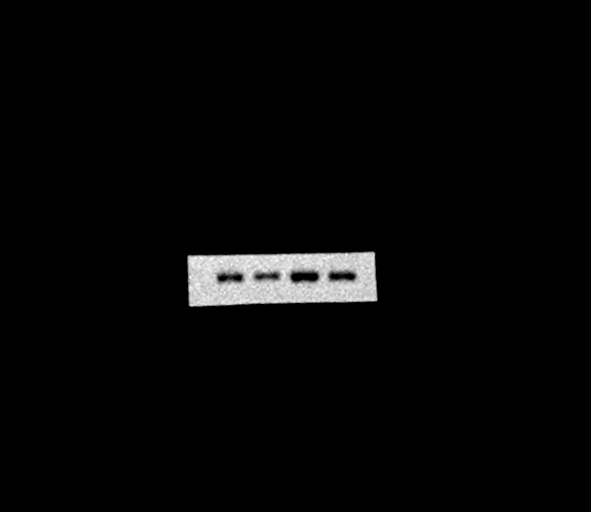


PCNA


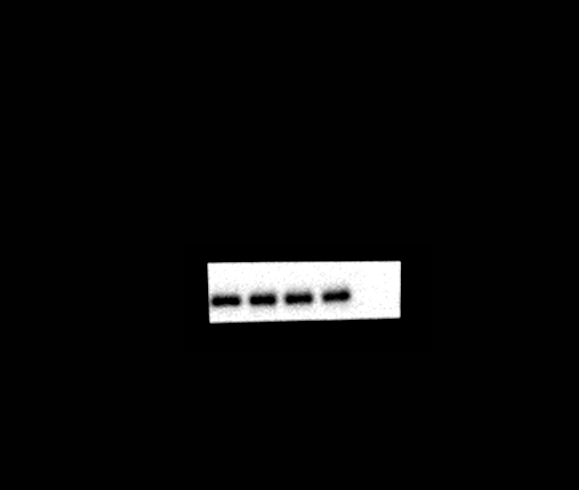


β-actin

Original Figure 8C


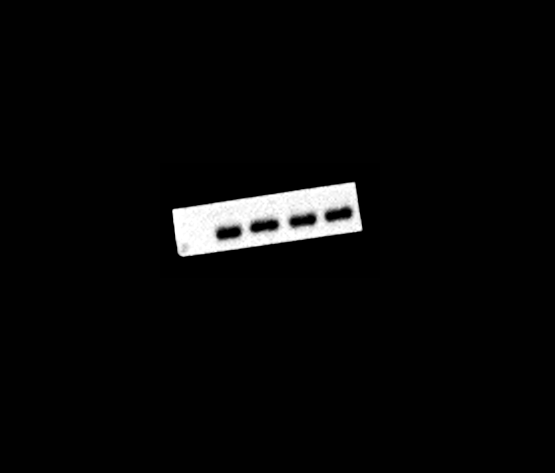


caspase-3


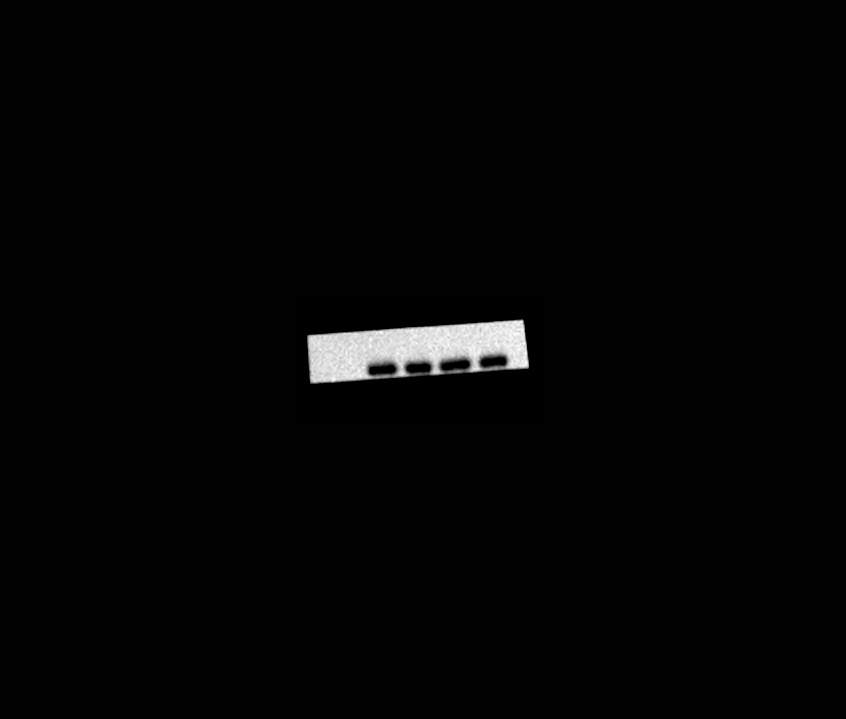


caspase-9


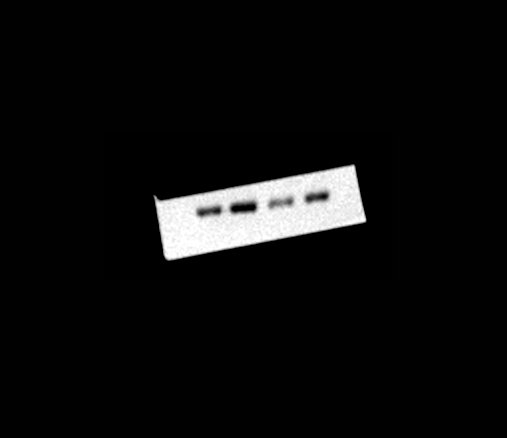


c-caspase-3


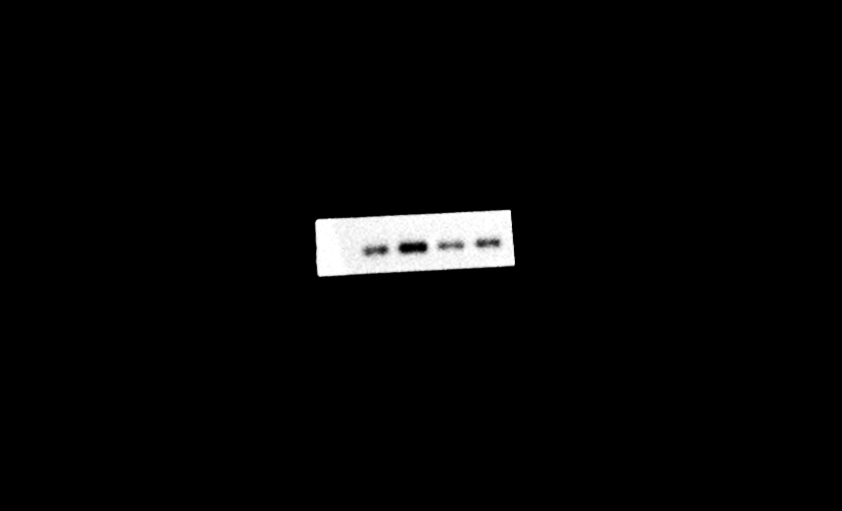


c-caspase-9


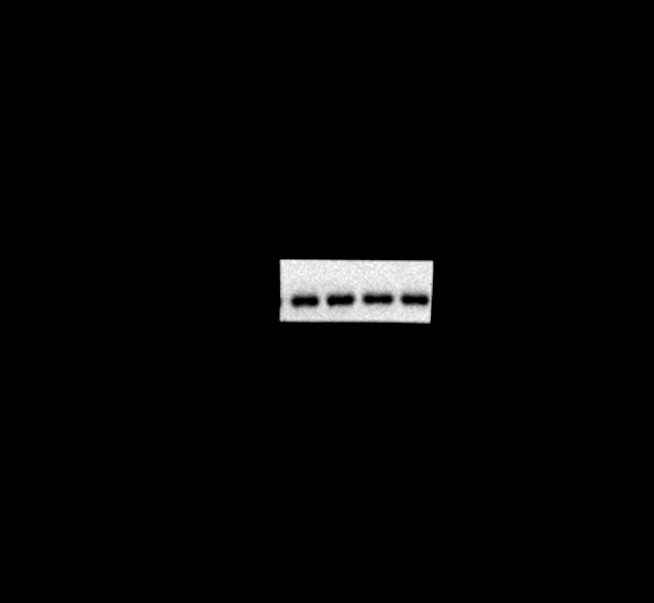


β-actin

Original Figure 8D


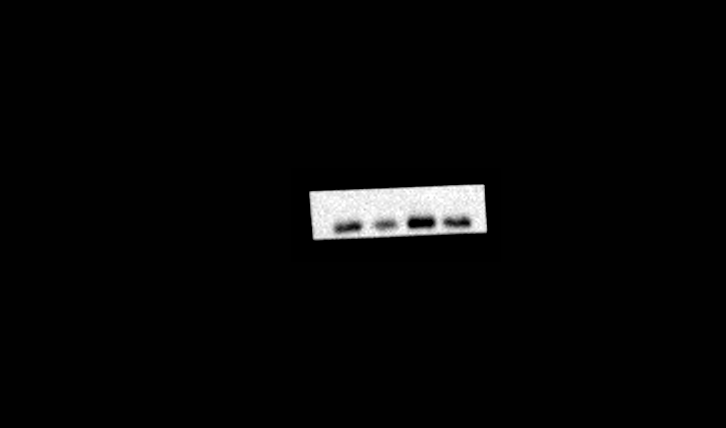


MMP-2


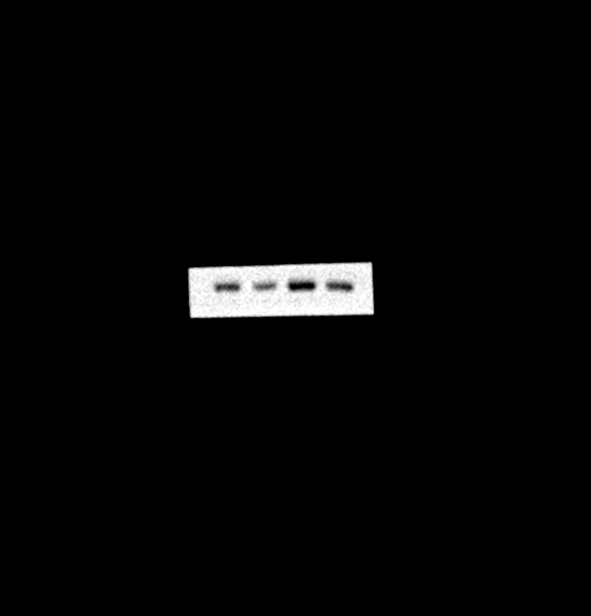


MMP-9


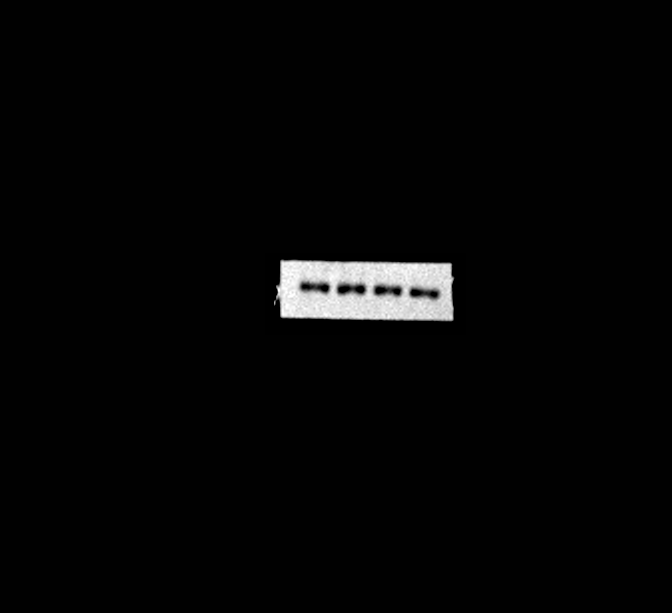


β-actin

Original Figure 8E


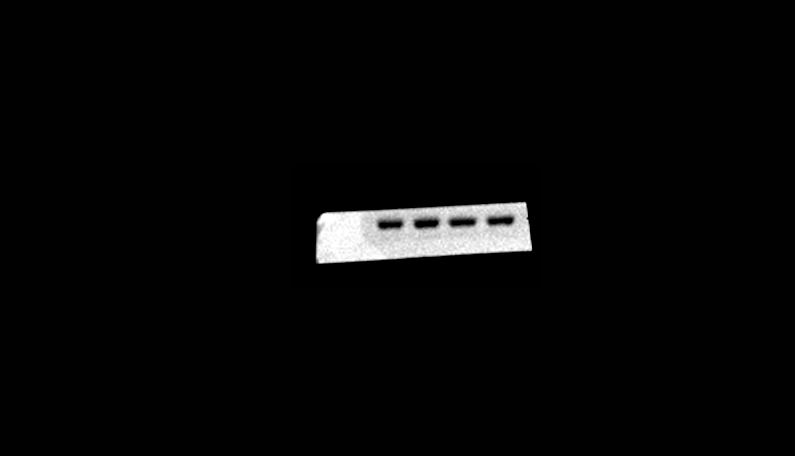


ERα


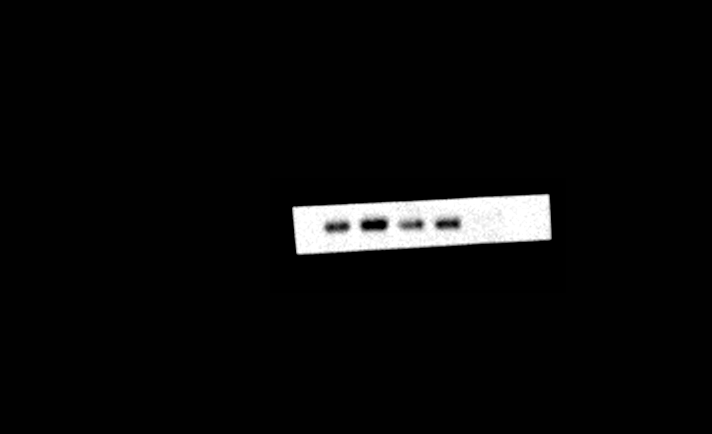


ERβ


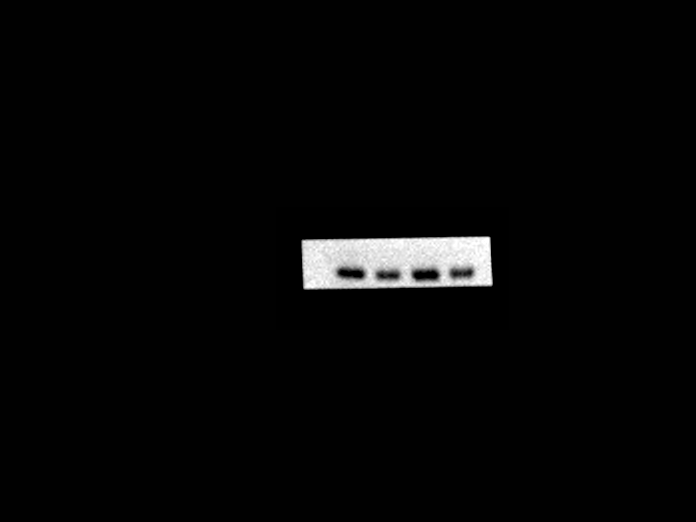


PES1


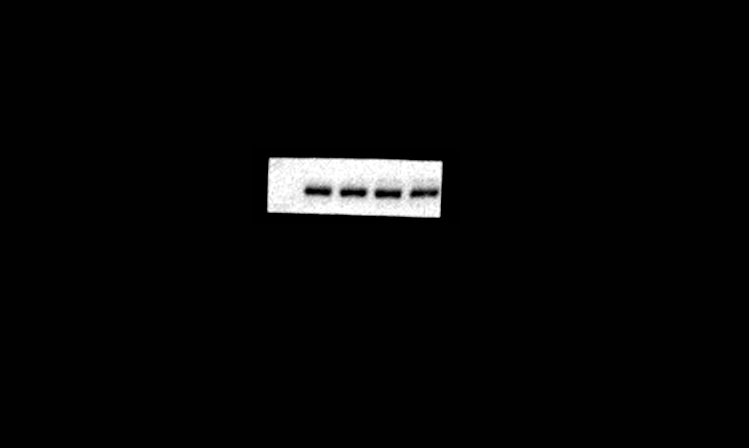


β-actin
